# Online forecasting for financial time series

**DOI:** 10.1007/s41060-026-01302-7

**Published:** 2026-10-16

**Authors:** Nikolas Michael, Mihai Cucuringu, Sam Howison

**Affiliations:** 1 Department of Statistics, University of Oxford, St Giles’ 24-29, Oxford, OX1 3LB UK; 2 Mathematical Institute, University of Oxford, Andrew Wiles Building 24-29, Oxford, OX2 6GG UK; 3 Oxford-Man Institute of Quantitative Finance, University of Oxford, Oxford, OX2 6GG UK; 4 The Alan Turing Institute, University of Oxford, 96 Euston Rd, London, NW1 2DB UK

**Keywords:** Financial time series, Time series analysis, Forecasting applications, Quantitative finance, Correlation

## Abstract

We introduce OFTER (online forecasting via temporal embedding regression), a time series forecasting pipeline tailored for financial problems centered on mid-sized multivariate time series. OFTER utilizes the non-parametric models of k-nearest neighbors and generalized regression neural networks, integrated with a dimensionality reduction component. To circumvent the curse of dimensionality, we employ a weighted norm based on a modified version of the maximal correlation coefficient. The pipeline we introduce is specifically designed for online tasks, has an interpretable output, and is able to outperform several state-of-the-art baselines. The computational efficacy of the algorithm, its online nature, and its ability to operate in low signal-to-noise regimes, render OFTER an ideal approach for financial multivariate time series problems, such as daily equity forecasting. Our work demonstrates that while deep learning models hold significant promise for time series forecasting, traditional methods carefully integrating mainstream tools remain very competitive alternatives with the added benefits of scalability and interpretability.

## Introduction

Time series are temporally structured data sets that play a central role in finance. For forecasting tasks, the temporal structure can be leveraged to improve performance but also to avoid look-ahead biases in the training phase. For univariate time series, parametric linear time series models such as the Autoregressive Integrated Moving Average (ARIMA) and the Autoregressive Conditional Heteroscedasticity (ARCH) have attained sufficiently good results in most applications of interest. While these algorithms can be extended to the setting of multivariate time series, the rapid rise in computing power in recent decades has rendered machine learning techniques an attractive alternative to traditional time series methods. An early comparison between different machine learning models was [1], where the authors concluded that the choice of method had a significant effect on performance.

Deep learning models have been a popular choice for multivariate time series forecasting, including Long-Short Term Memory (LSTM) [2], Bidirectional LSTM [3] and Gated Recurrent Unit [4] (GRU). Various expansions of such deep learning models have been proposed in recent years, such as DeepAR [5] and Temporal Fusion Transformers [6], which generally achieve state-of-the-art performances across various domains. However, such approaches often employ thousands of trainable parameters, which can be difficult to train with a limited number of observations, thus leading to degraded out-of-sample forecasting performance. Despite such advancements in deep learning, simple regression-based models are still widely used and sought after. This is especially true for mid-low frequency financial tasks, such as predicting daily returns, volumes, and realized volatilities, partly due to the complexity, unpredictability and constantly shifting dynamics of the financial markets. For example, ordinary least squares (OLS) is still widely used for both forecasting and interpretive statistical analysis due to its simplicity, closed-form solution, and the fact that it requires no choice of hyperparameters. Its regularized versions, such as the LASSO and the elastic net are some of the most widely utilized regression models in finance, as they require only a few hyperparameters.

On the other hand, various recent lines of work have demonstrated the merit of combining traditional time series models and machine learning models, such as [7–11]. In this paper, we consider one of the simplest types of hybrid model, which builds on the results of a univariate ARIMA model and enhances it by performing a novel regression method on the residuals of an ARIMA model.

### Non-parametric models and dimensionality reduction

Two simple models that have been relatively overlooked by the machine learning community, are k-nearest neighbors (kNN) and generalized regression neural networks (GRNN), both of which are simple, non-parametric, fast methods requiring only a single hyperparameter. This mainly stems from the curse of dimensionality, which affects both techniques. GRNNs have been utilized primarily in engineering applications, such as [12] for logo recognition, [13] for forecasting feeding patterns, and [14] for modeling the pile-bearing capacity of soil. An overview of its different applications for dynamical systems has been pursued by [15]. In this work, we aim to circumvent the curse of dimensionality that these two methods suffer from by using the following two techniques. Firstly, instead of the Euclidean norm employed by kNN and GRNN, we consider a thresholded weighted norm that is driven by the feature’s predictive potential. Secondly, before employing these well-known models, we consider a dimensionality reduction component, which is by far the most common remedy for the curse of dimensionality.

Dimensionality reduction is one of the main unsupervised machine learning paradigms, with the past couple of decades having witnessed seminal contributions in terms of nonlinear dimensionality reduction methods, such as locally linear embedding (LLE) [16], ISOMAP [17], Hessian LLE [18], Laplacian eigenmaps [19], and diffusion maps [20]. In more recent years, deep learning models have also been increasingly used as a means of dimensionality reduction, such as the class of autoencoders [21], which can be seen as a nonlinear extension of PCA. Performing dimensionality reduction via the use of autoencoders before employing a kNN technique, [22] has shown a significant improvement in performance over kNN for classification. Partial least squares are a popular method of incorporating information from the target into the initial features, during the embedding stage. More recently, [23] achieved improvements in the performance of PCA by scaling each factor based on its predictive slope with the target.

### Uses in financial applications

Examples of dimensionality reduction use cases in time series forecasting tasks include [24], who used a truncated diffusion map to construct a global manifold for the slow dynamics of a reduced kinetic model, and [25] who utilizes manifold learning techniques in conjunction with various supervised learning models to build a profitable strategy for the Forex market. Along the same lines, [26] proposed a hybrid approach integrating the Midas-RV model and PCA to achieve better results in forecasting the volatility of crude oil futures in the Chinese market. In this paper, we consider the forecasting of daily returns and traded volumes for ETF instruments. Being able to accurately predict next-day volumes is of interest to practitioners and especially to market makers. Previous works include [27], who showed how search engine data can be leveraged to forecast future trading volumes. Similarly to our work is [28], who utilizes support vector machines to predict the daily volume percentages with promising performance, in the spirit of [29] who combine SVM and LSTM after residualizing with an AR model.

### Main contribution OFTER - a novel integrated pipeline

We consider the task of one-step time series forecasting and propose an approach that integrates ARIMA models, dimensionality reduction, and simple non-parametric methods into a single pipeline, which we denote as *online forecasting via temporal embedding regression (OFTER)*. Such a pipeline is of great interest to practitioners working with mid-sized data sets that are low signal-to-error in nature, with no significant seasonality patterns, with low frequency trading strategies being a common example that we explore.

To alleviate the curse of high dimensionality and enhance our dimensionality reduction component, we conduct feature selection on the embedding features based on the concept of *maximal correlation*. We apply OFTER to the financial tasks of forecasting next-day volume and returns by building an embedding based on various features of 10 exchange traded funds (ETFs). Through an extensive set of numerical simulations on both synthetic and real data, we demonstrate that OFTER provides a powerful alternative to other mainstream machine learning models for mid-sized data sets, even performing better than established time series-tailored deep learning models. In particular, it can work effectively in low signal-to-noise regimes for mid-sized data sets, while still being computationally efficient.

Furthermore, our proposed methodology provides the following additional benefits.

- Once an embedding has been computed, the algorithm can easily perform out-of-sample extensions and incorporate newly arrived time observations. Therefore, OFTER can naturally be viewed as an online algorithm that operates efficiently in an online setting.
- Certain configurations of our method fall under the unsupervised learning setting, as the embedding is independent of the target. In other words, the same embedding can potentially be employed to forecast different target variables of interest, and thus OFTER is amenable to extensions to the multi-target setting.
- Our method can further provide an avenue for change-point detection and anomaly detection tasks, which can be seen as natural byproducts. A previous or newly arrived observation without any local neighboring points in the embedding, could indicate either an extreme measurement or a change-point in the time series.

### Paper structure

The rest of this paper is structured as follows. In Sect. 2, we provide an overview of some of the essential concepts used in the proposed pipeline, including the kNN and GRNN algorithms, principal component analysis, and maximal correlation. In Sect. 3, we introduce our proposed methodology, along with its main components and their integration into an algorithm. In Sect. 4, we analyze the performance of OFTER in both synthetic experiments and real-world data. We also discuss the additional properties of OFTER in Sect. 5. Finally, we conclude in Sect. 6.

## Preliminaries and related works

Consider a *d*-dimensional time series represented as a matrix $X = \left( x_1,\dots ,x_T \right) \in \mathbb {R}^{T \times d}$ with rows denoting observations for *T* time points, which we refer to as the feature matrix. Assume for the rest of this paper that we are interested in forecasting a single univariate target variable, which we denote by $y = \left( y_1,\dots ,y_T \right) \in \mathbb {R}^{T}$ and refer to as the target variable. Furthermore, denote by $x' \in \mathbb {R}^{d}$ and $y' \in \mathbb {R}$, the value of the features and the target variable, respectively, associated with a new observation. We denote by $e_d$ the all-ones vector of length *d*. In terms of matrix indexing, we generally denote the (*i*, *j*) entry of *X* by $X_{i,j}$, the submatrix of *X* including rows starting from $t_1$ up to $t_2$ by $X_{t_1:t_2}$, i.e $\left( x_{t_1},\dots ,x_{t_2} \right)$ and by $X_{t_1:t_2,j}$ the $j^{th}$ column of $X_{t_1:t_2}$.

### kNN and GRNN

kNN is a non-parametric supervised learning algorithm that dates back to the work of Fix and Hodges in 1951 [30] and has grown to become one of the most popular machine learning techniques. The main idea is that a similarity of values in the feature space implies a similarity in the values for the target variable. Denote by $x_{(1)}, x_{(2)}, \dots , x_{(T)}$ a reordering of the training samples such that

1 $$\begin{aligned} \Vert x_{(1)} - x'\Vert \ \le \ \cdots \ \le \ \Vert x_{(T)} - x'\Vert , \end{aligned}$$

where $$\Vert \cdot \Vert $$ denotes the Euclidean norm. The set of the *k*-nearest neighbors of the new point $x^{\prime }$ is defined as

2 $$\begin{aligned} \text {NN}_k(x') = \{ i \in \{ 1,\dots ,T \} : || x_{(i)} - x' || \le || x_{(k)} - x' || \}. \end{aligned}$$

Based on $$\text {NN}_k(x')$$, the forecast for $y^{\prime }$ is computed as

3 $$\begin{aligned} \hat{y} = \frac{1}{k} \sum _{i \in \text {NN}_k(x') } y_i. \end{aligned}$$

A closely related method is GRNN, introduced in [31], based on the Nadaraya-Watson kernel regression estimator [32, 33]. When forecasting $y^{\prime }$, GRNN attaches a weight to each of the previous time series entries, determined by their distance to $x^{\prime }$. In particular, define the weight $$w_i$$ of the $$i^{th}$$ observation as

4 $$\begin{aligned} w_i = \frac{\exp \left( \frac{-|| x_i - x' ||^2}{h} \right) }{\sum \limits _{j=1}^{T} \exp \left( \frac{-|| x_j - x' ||^2}{h} \right) }, \end{aligned}$$

where *h* is a bandwidth hyperparameter to be optimized. The prediction for the response of a new observation $\hat{y}$ is then simply the weighted average of the responses of the training observations, defined as

5 $$\begin{aligned} \hat{y} = \sum _{i=1}^T w_i y_i. \end{aligned}$$

Consider the observations $(x_1,y_1),\dots ,(x_T,y_T)$, to be realizations of an $\mathbb {R}^{d+1}$ random vector, and let *f* denote the corresponding true underlying density of the random variable. The theoretical motivation of GRNN stems from the Parzen estimators [31], used to estimate unknown probability density functions *f* as

6 $$\begin{aligned} \hat{f}_h(x',y')  &   = \frac{1}{T} \frac{1}{\left( \pi h \right) ^{\frac{d+1}{2}}} \sum _{i=1}^T \text {exp} \!\left[ -\frac{(y'-y_i)^2}{h} \right] \nonumber \\  &   \quad \times \text {exp} \!\left[ -\frac{(x'-x_i)^T (x'-x_i)}{h} \right] . \end{aligned}$$

If *h* varies with *T* such that $\lim _{T \rightarrow \infty } h(T) = 0$ and $\lim _{T \rightarrow \infty } Th^d(T) = 0$, these estimators are shown to be consistent, i.e

7 $$\begin{aligned} plim_{T \rightarrow \infty } \hat{f}_{h(T)}(x',y') = f(x',y'), \ \ \forall (x',y') \in \mathbb {R}^{d+1}. \end{aligned}$$

Informally, [31] argues that if $\hat{f}(x,y) \approx f(x,y)$, then

8 $$\begin{aligned} \begin{aligned} \hat{y} = \sum _{i=1}^T w_i y_i&= \int y' \frac{\hat{f}(x,y')}{ \int \hat{f}(x,y) dy} dy' \\&\approx \int y' \frac{f(x,y')}{ \int f(x,y) dy} dy' = \mathbb {E} \left[ y' | x' \right] . \end{aligned} \end{aligned}$$

We caution the reader that, despite GRNN bearing “Neural Network" in its name, it is quite different from other popular variants. Specifically, aside from the width hyperparameter, there are no ad-hoc hyperparameters to be chosen or optimized. Furthermore, it is a single-pass algorithm, as the weights are not optimized. Thus, unlike other neural network structures, it is much faster and easier to train but lacks the expressive power of neural networks.

### Dimensionality reduction

Both kNN and GRNN suffer from the so-called *curse of dimensionality* which refers to the phenomenon that for certain methods, the amount of data points required to estimate an unknown function with a certain accuracy, grows exponentially with the dimensionality of the data set.^1^ To alleviate this issue, kNN or GRNN is often applied after projecting the data points from $\mathbb {R}^d$ into a lower-dimensional space $\mathbb {R}^{p}$ (p≤d), while somewhat retaining the structure of the original data set. By far the most popular such method is principal component analysis (PCA), which applies a linear transformation to the original features, chosen to maximize the variance of the projected features in the newly reduced space while ensuring the resulting features remain uncorrelated with each other.

Denote by *C*(*X*) the covariance matrix of *X*, and its eigen-decomposition by $C(X) = U \Lambda U^T$, where Λ is a diagonal matrix capturing the $\lambda _1 \ge \dots \ge \lambda _d \ge 0$ eigenvalues of *C*(*X*), and the columns of *U* are given by the $u_1,\dots ,u_d$ orthonormal eigenvectors (in the same order as their corresponding eigenvalues). The principal component scores are given by the columns of *XU*, with the variance of each of the new components given by its corresponding eigenvalue (component *i* has variance $\lambda _i$). Selecting the first *p* components retains a proportion of the total variance in the feature matrix equal to $\sum _{i=1}^p \lambda _i / \sum _{i=1}^d \lambda _i$. Given the proportion of variance to retain $\delta \in (0,1)$, we set

9 $$\begin{aligned} p = \text {min} \left\{ i: \frac{\sum \limits _{k=1}^i \lambda _k}{\sum \limits _{k=1}^d \lambda _k} \ge \delta \right\} . \end{aligned}$$

We remark that random-matrix theory based techniques could also be employed for delimiting the informative part of the spectrum from the bulk of the eigenvalues that correspond to the noise component [34]. Note that before calculating *C*(*X*), we ensure that *X* is of full rank by removing any columns of *X* that result in rank deficiency.^2^ Before applying PCA, we standardize *X* (subtracting by each feature its mean and dividing by its standard deviation). We shall denote the transformed features as $\tilde{X} = XU$.

### Maximal correlation

Consider two univariate random variables denoted as $V_1$ and $V_2$. Loosely stated, the correlation between $V_1$ and $V_2$ is a measure of their association. Popular types of correlation include the Pearson, Spearman, and Kendall correlations. Another type is the maximal correlation (see e.g [35]) defined as

10 $$\begin{aligned} \underset{\phi ,\psi \in \mathcal {F}}{\text {argmax}} \ \text {Cor}(\phi (V_1),\psi (V_2)), \end{aligned}$$

where $\mathcal {F}$ is a suitable function class and $\text {Cor}$ is the Pearson correlation defined as

11 $$\begin{aligned} \text {Cor}(V_1,V_2) = \frac{\mathbb {E} \left[ V_1 V_2 \right] - \mathbb {E} \left[ V_1 \right] \mathbb {E} \left[ V_2 \right] }{\sqrt{\mathbb {E} \left[ V_1^2 \right] - \mathbb {E} \left[ V_1 \right] ^2} \sqrt{\mathbb {E} \left[ V_2^2 \right] - \mathbb {E} \left[ V_2 \right] ^2}}. \end{aligned}$$

A solution to the problem of determining maximal correlation can be computed by optimizing the functions ϕ and ψ iteratively. We consider a related but simpler problem, where we aim to calculate

12 $$\begin{aligned} \underset{\phi \in \mathcal {F}}{\text {argmax}} \ \text {Cor}(\phi (V_1),V_2). \end{aligned}$$

The reason for considering Eq. (12) instead of Eq. (10), is that in a regression setting, we are only interested in transforming the feature, rather than the target. By solving Eq. (10), one can transform the features in the embedding space so as to be maximally correlated to the target.

We consider a solution in a restricted function class $\mathcal {F}$.^3^ In particular, we consider the class of Bernstein polynomials (see e.g [36]), which are defined as

13 $$\begin{aligned} b_{\nu ,n}(x) = \left( {\begin{array}{c}\nu \\ n\end{array}}\right) x^\nu (1-x)^{n-\nu }, \quad \nu = 0, 1, \dots , n. \end{aligned}$$

The choice of this basis is justified via the Stone–Weierstrass theorem.^4^ Bernstein polynomials have been widely used, especially in the context of Bezier curves [37], and B-splines [38], often arising in the development of modeling tools in computer-aided geometric design.

For the solution of Eq. (12), we assume that we observe vectors $v_1$ and $v_2$, which capture *N* observations of the random variables $V_1$ and $V_2$, respectively. Denote by $\bar{v}_1$ and $\bar{v}_2$ the mean of $v_1$ and $v_2$, respectively. Similarly denote by $\sigma _{v_1}$ and $\sigma _{v_2}$ standard deviation of $v_1$ and $v_2$, respectively. Consider the function basis $\left( \phi _1, \dots , \phi _K \right)$, where $\phi _i(x) = b_{K,i}(x)$ and define the N×K matrix Φ s.t $\Phi _{i,j} = \phi _j(v_{1,i})$. We assume Φ is centered, i.e $\Phi ^T e_N = {\textbf {0}}$.

With the function basis defined, we consider the function class

14 $$\begin{aligned} \mathcal {F} = \left\{ \sum _{k=1}^{K} c_k \phi _k : c_1,\dots ,c_K \in \mathbb {R} \right\} , \end{aligned}$$

so that for $f \in \mathcal {F}$, $f(v_{1,i}) = \sum _{k=1}^{K} c_k \phi _k(v_{1,i}),$ and problem Eq. (12) reduces to finding the suitable vector *c*.

To simplify calculations, we also require the transformed vector to be standardized, i.e $c^T \Phi ^T e_N =0, \frac{1}{N-1} c^T \Phi ^T \Phi c = 1$. Thus, we aim to solve

15 $$\begin{aligned} \underset{c \in \mathbb {R}^K,\ c^T \Phi ^T e_N =0, \ c^T \Phi ^T \Phi c = N-1}{\text {argmax}}\text {Cor}(\Phi c,v_2). \end{aligned}$$

Note that

16 $$\begin{aligned} \begin{aligned} \text {Cor}(\Phi c,v_2)&= \frac{\text {Cov}(\Phi c,v_2)}{\sigma _{v_2} \sigma _{v_1}} \\&= \frac{\frac{(\Phi c)^T v_2}{N-1} - \left[ \frac{(\Phi c)^T e_N}{N} \right] \left[ \frac{v_2^T e_N}{N} \right] }{\sigma _{v_2} \sigma _{v_1}}, \end{aligned} \end{aligned}$$

and thus, under the constraints of Eq. (15), we have

17 $$\begin{aligned} \text {Cor}(\Phi c,v_2) = \frac{(\Phi c)^T v_2}{(N-1) \sigma _{v_2}}, \end{aligned}$$

for which a solution can be found by considering the Lagrangian

18 $$\begin{aligned} L = \frac{ c^T \Phi ^T v_2}{(N-1) \sigma _{v_2}} - \lambda (c^T \Phi ^T e_N) - \mu (c^T \Phi ^T \Phi c - (N-1)). \end{aligned}$$

Extremizers of the above Lagrangian satisfy

19 $$\begin{aligned} \mu = \frac{ c^T \Phi ^T v_2}{2(N-1)^2 \sigma _{v_2}}, \text { and} \end{aligned}$$

20 $$\begin{aligned} \lambda = \frac{\bar{v}_2}{(N-1) \sigma _{v_2}}. \end{aligned}$$

Substituting back to Eq. (18), and writing $z = \Phi ^T v_2$, $\bar{z} = z - \bar{v}_2 \Phi ^T e_N$ and $A = \frac{\Phi ^ T \Phi }{N-1}$, we reduce the problem to solving

21 $$\begin{aligned} \bar{z} = \left( c^T z \right) A c. \end{aligned}$$

By considering a solution of the form $c = \tau A^{-1} \bar{z}$ for $\tau \in \mathbb {R}$, we arrive at the solution

22 $$\begin{aligned} \tau = \frac{1}{\sqrt{\bar{z}^T (A^{-1})^T z}}. \end{aligned}$$

Overall, the solution to Eq. (15) is given by

23 $$\begin{aligned} c&= \frac{A^{-1}\bar{z}}{\sqrt{\bar{z}^T (A^{-1})^T z}} \nonumber \\  &= \frac{\sqrt{N-1}\, (\Phi ^T \Phi )^{-1}\Phi ^T \left( v_2-\bar{v}_2 e_N\right) }{\sqrt{ \left( v_2-\bar{v}_2 e_N \right) ^T \Phi \left[ (\Phi ^T \Phi )^{-1}\right] ^T \Phi ^T v_2 }}. \end{aligned}$$

We refer to the correlation between the target *y* and the transformed *x* as the *one-sided maximal correlation* (OSMC) of x and *y*, as

24 $$\begin{aligned} \text {OSMC}_K(x,y) = \text {Cor}(\Phi c,y), \end{aligned}$$

where Φ and *c* depend on *x* and the choice of the number of basis functions used *K*. In Section F, we prove the consistency of the transformation $c_N$ and show that it has a convergence rate of $O_p(N^{-1/2})$.

## Methodology

**Fig. 1:**
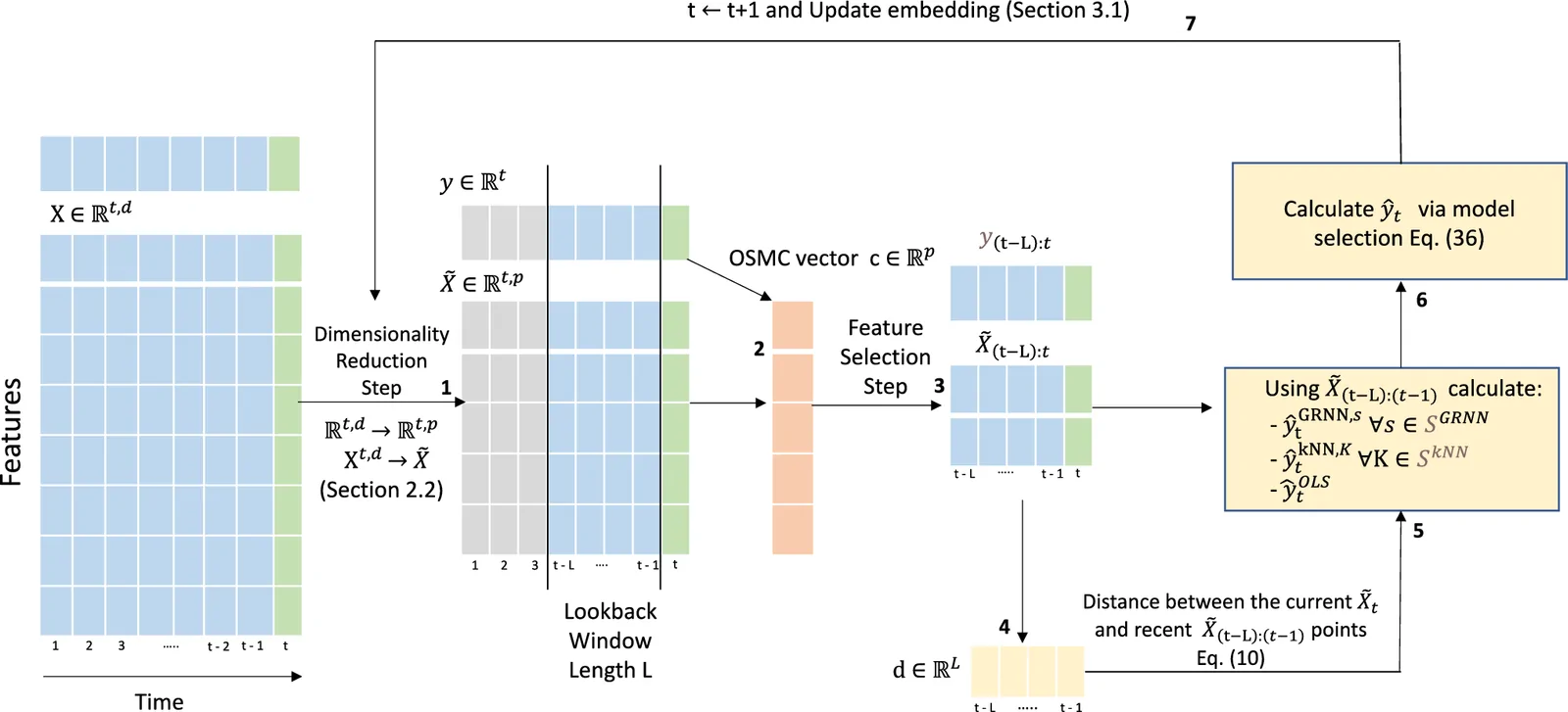
Pictorial illustration of OFTER, where the main components of its iterative part are displayed. Note that some components are omitted to simplify the diagram. **(1)** In this step, the feature matrix $X \in \mathbb {R}^{n,d}$ is transformed to $\tilde{X} \in \mathbb {R}^{n,p} \ \ (p < d)$ using PCA. **(2)** Each entry of the vector *c* is calculated as $c_j = \text {OSMC}(\tilde{X}_{1:L_0, j },y_{1:L_0})$, with OSMC as formulated in Sect. 2.3. **(3)** Each entry of the vector *v* is calculated as $v_j = \frac{c_j^2 \mathbb {1}(|c_j | \ge c_{\text {min}})}{\sum \limits _{l=1}^{p + |I^{\text {Original}}|} c_l^2 \mathbb {1}(| c_l | \ge c_{\text {min}})}$, as is explained in Sect. 3.3. In other words, features whose correlation with the target is low are discarded. **(4)** The distance between observations of time points *j* and *t* is calculated as $d_{j,t} = || \tilde{X}_{ t} - \tilde{X}_{ j}||_v$, where $||.||_v$ is later defined in Eq. (44). This is calculated for j=t-L,⋯,t-1 where *L* is the lookback window. **(5)** All the different forecasts from GRNN, kNN, and OLS are calculated using $\tilde{X}_{(t-L):(t-1)}$, and the distance vector *d*. **(6)** All the individual forecasts are combined into $\hat{y}_t$. In particular, the forecast of the highest performing model is selected as explained in Sect. 3.2. **(7)** The PCA embedding is updated using the methodology described in Sect. 3.1. In particular, the eigenvalues and eigenvectors of the covariance matrix is updated

Figure 1 is a pictorial diagram of the main components of the OFTER pipeline, each of which is described in detail in the remainder of this section.

### Online update of the embedding

In order for the algorithm to run in an online setting, the embedding needs to be updated as new data arrives. The initial embedding is calculated based on the first $L_0 < T$ entries, and then updated at every time step. However, re-computing the embedding at every single iteration can be quite expensive (in fact, of complexity $O(nd^2 + d^3)$ at each step^5^). For PCA, there exists a computationally efficient approach that updates *U* and Λ. In particular, if $C(X_t)$ denotes the covariance matrix built from the first *t* data points, one can compute the eigenvectors of the updated covariance

25 $$\begin{aligned} C(X_{1:t+1}) = \frac{t-1}{t} C(X_{1:t}) + \frac{1}{t} || x_{t+1} ||^2 \frac{x_{t+1}}{|| x_{t+1} ||} \frac{x_{t+1}^T}{|| x_{t+1} ||}, \end{aligned}$$

by using the previously computed eigenvectors of $C(X_{1:t})$ (we assume centered data for now). The online algorithm proposed in [39] achieves just that, with a complexity of $O(p^2 d)$ at each iteration (recall that *p* denotes the chosen dimension of the embedding).

The above is an instance of a more general problem, which is the rank-one update of an eigensystem [40]. Consider again the symmetric matrix *A*, whose eigen-decomposition is known, with $\lambda _1 \ge \dots \ge \lambda _d$ denoting its eigenvalues, and $u_i$ be the eigenvector corresponding to $\lambda _i, \forall i \in \{ 1,\dots ,d \}$ such that $A = U \Lambda U^T$. The task at hand is to evaluate the eigensystem of $A + \rho v v^T$, by using the known eigensystem of *A*. As shown by [40], the eigenvalues of $A + \rho v v^T$ are the roots $\kappa _1,\dots ,\kappa _d$ of the *secular equation*

26 $$\begin{aligned} 1 + \rho \sum _{i=1}^d \frac{z_i^2}{\lambda _i - \kappa } = 0, \ \text {where} \ z = U^T v. \end{aligned}$$

Once the updated eigenvalues are computed, each updated eigenvector $u_k$ can be calculated as

27 $$\begin{aligned} u_k = \frac{U \left( \Lambda - \kappa _k I \right) ^{-1} z}{||U \left( \Lambda - \kappa _k I \right) ^{-1} z || }. \end{aligned}$$

Going a step further, one could reduce computations by only updating the first few eigenvalues. A good approximation of the solution is given by [41], who shows how this can be achieved by solving

28 $$\begin{aligned} 1 + \rho \left( \sum _{i=1}^p \frac{z_i^2}{\lambda _i-\kappa } + \frac{1-\sum \limits _{i=1}^p z_i^2}{\mu -\kappa } \right) = 0. \end{aligned}$$

The solutions of this equation are shown to approximate the first *m* eigenvalues, with an error of the order $O(\underset{m+1 \le i \le p}{\text {max}} |\lambda _i - \mu |)$. Note that μ is another parameter to be chosen, and should be made so as to minimize $O(\underset{m+1 \le i \le p}{\text {max}} |\lambda _i {-} \mu |)$, for example $\mu {=} \text {median}(\lambda _{m+1},\dots ,\lambda _p)$. The parameter *p* will have already been chosen at an earlier part of the algorithm using Eq. (9), and does not change during the update step. While there are various existing techniques to solve equation Eq. (28), we employ the convexifying method described in [42], adopting the earlier work of [40].

Note that initially the feature matrix is standardized based on the initial $L_0$ entries (i.e we standardize $X_{(1:L_0)}$). Denote the mean vector of $X_{1:t}$ by $\mu _t \in \mathbb {R}^d$, and the correctly centered $X_{1:t}$ by $\bar{X}_{1:t} \in \mathbb {R}^d$. Assuming that $X_{(1:t-1)}$ has been centered by $\mu _{t-1}$, we need to re-center based on $\mu _{t}$. Following [39], the update takes the form $\bar{X}_{1:t} = X_{1:t} + e_t (\mu _{t-1}-\mu _{t})^T$. It turns out that we can adjust for the re-centering, via two more rank-one updates of the covariance matrix, by expressing $\bar{X}^T_{1:t} \bar{X}_{1:t}$ in the following way.

$$\begin{aligned} \bar{X}^T_{1:t} \bar{X}_{1:t}&= X_{1:t}^T X_{1:t} + X_{1:t}^T e_N \delta ^T + \delta \, e_N^T X_{1:t} + t\, \delta \delta ^T \\&= X_{1:t}^T X_{1:t} + \begin{bmatrix} \delta&X_{1:t}^T e_N \end{bmatrix} \begin{bmatrix} t &  1 \\ 1 &  0 \end{bmatrix} \begin{bmatrix} \delta ^T \\ e_N^T X_{1:t} \end{bmatrix} \\&= X_{1:t}^T X_{1:t} + \begin{bmatrix} \delta _\mu&X_{1:t}^T e_N \end{bmatrix} \begin{bmatrix} \frac{\rho _1}{\sqrt{1+\rho _1^2}} &  \frac{\rho _2}{\sqrt{1+\rho _2^2}} \\ \frac{1}{\sqrt{1+\rho _1^2}} &  \frac{1}{\sqrt{1+\rho _2^2}} \end{bmatrix} \\&\qquad \times \begin{bmatrix} \rho _1 &  0 \\ 0 &  \rho _2 \end{bmatrix} \\&\qquad \times \begin{bmatrix} \frac{\rho _1}{\sqrt{1+\rho _1^2}} &  \frac{\rho _2}{\sqrt{1+\rho _2^2}} \\ \frac{1}{\sqrt{1+\rho _1^2}} &  \frac{1}{\sqrt{1+\rho _2^2}} \end{bmatrix}^T \begin{bmatrix} \delta _\mu ^T \\ e_N^T X_{1:t} \end{bmatrix} \\&= X_{1:t}^T X_{1:t} + \begin{bmatrix} \frac{\rho _1\delta + X_{1:t}^T e_N}{\sqrt{1+\rho _1^2}}&\frac{\rho _2\delta + X_{1:t}^T e_N}{\sqrt{1+\rho _2^2}} \end{bmatrix} \begin{bmatrix} \rho _1 &  0 \\ 0 &  \rho _2 \end{bmatrix} \\&\qquad \times \begin{bmatrix} \frac{\rho _1\delta + X_{1:t}^T e_N}{\sqrt{1+\rho _1^2}} \\ \frac{\rho _2\delta + X_{1:t}^T e_N}{\sqrt{1+\rho _2^2}} \end{bmatrix} \\&= X_{1:t}^T X_{1:t} + \rho _1 b_1 b_1^T + \rho _2 b_2 b_2^T. \end{aligned}$$

where in the above

29 $$\begin{aligned} \delta _\mu = \mu _{t-1}-\mu _t, \end{aligned}$$

30 $$\begin{aligned} \rho _1 =\frac{t - \sqrt{t^2 + 4}}{2}, \end{aligned}$$

31 $$\begin{aligned} \rho _2 = \frac{t + \sqrt{t^2 + 4}}{2}, \end{aligned}$$

32 $$\begin{aligned} b_1 = \frac{\rho _1 (\mu _{t-1}-\mu _{t}) + X_{1:t}^T e_N}{\sqrt{1 + \rho _1^2}}, \end{aligned}$$

33 $$\begin{aligned} b_2=\frac{\rho _2 (\mu _{t-1}-\mu _{t}) + X_{1:t}^T e_N}{\sqrt{1 + \rho _2^2}}. \end{aligned}$$

### Choice of hyperparameters

The hyperparameter *k* of kNN in Eq. (5) and the smoothing parameter hyperparameter *h* in Eq. (4) are both of crucial importance to the performance of their respective algorithms. Notice that, as $h \rightarrow \infty$, $w_i \rightarrow 1/T$ (Eq. (4)) for all *i*. On the other hand, as h→0, GRNN becomes equivalent to $1\text {NN}$ (kNN with k=1). To demonstrate the effect of the smoothing parameter on the predictive task, we consider the following toy model

34 $$\begin{aligned} y_t = 0.5 + \cos \left( \frac{\pi }{64} t \right) + \epsilon _t, \text {where } \epsilon _t \sim N(0,\sigma ^2). \end{aligned}$$

**Fig. 2:**
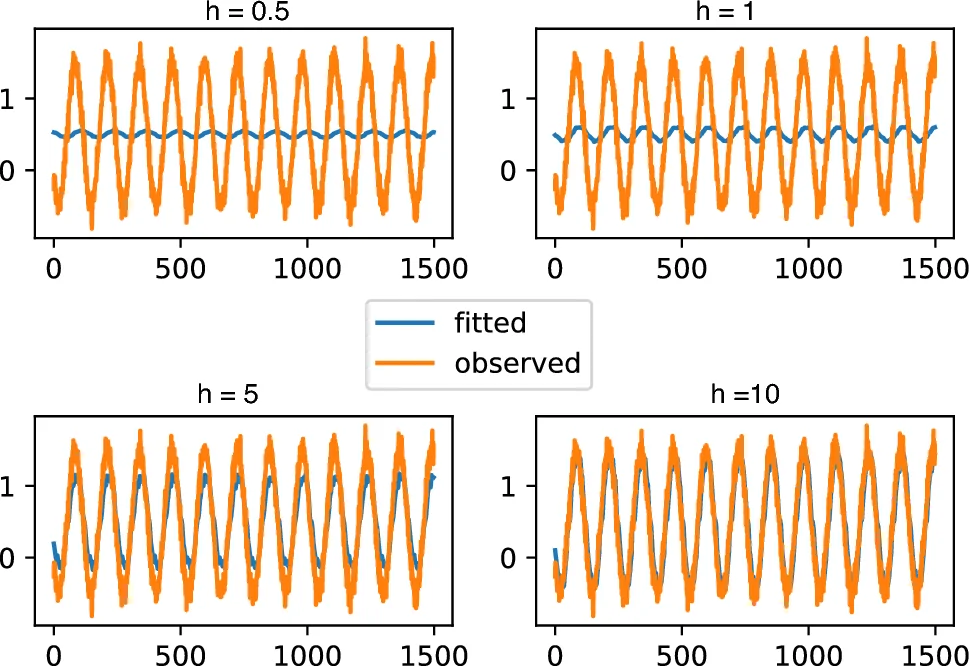
Visualization of the forecasted values when using GRNN with different smoothing parameters for the model Eq. (34). In a high signal-to-noise problem like this, as *h* becomes larger GRNN becomes almost perfectly capable of forecasting future values

In Fig. 2, we demonstrate how the choice of the *h* affects the nature of the obtained fit. For low values of *h*, the obtained fit is close to giving constant fitted values equal to the mean, while for high values of *h*, the obtained fit matches the observed data points almost perfectly.^6^

To choose a suitable value of *h*, [31] proposes the use of the holdout method, where the optimal parameter is found via calculating a loss function by removing a sample point at a time and taking the average. More modern applications use K-fold validation instead, and these methods can be modified for time series data (see [43] for a discussion on the different approaches).

To remove any scale dependencies, we re-parameterize *h* as

35 $$\begin{aligned} h = \frac{\text {median}(\{ || x_j - x' || \}_{j=1}^T)}{s}. \end{aligned}$$

Dynamic Selection Since this is an online algorithm, we could update the hyperparameters in an online manner as well. Instead of choosing a single value from a candidate set, we use each of the candidate values for the hyperparameter to fit a different model, updating the performance of all models at each time point. The model with the highest performance up to the previous time point is chosen for the forecast of the current time point, as is done in dynamic model selection [44, 45]. The region of competence includes the last $L_D$ time points (we refer to this as the lookback window).^7^

The choice of the parameter *s* can be performed by the use of a loss function. If the fitted value corresponding to parameter *s* for day *t* is $\hat{y}^s_t$, the forecast $\hat{y_t}$ is calculated as

36 $$\begin{aligned} \hat{y}_t&= \sum _{s=1}^{|S|} \eta _t^s \hat{y}_t^s, \quad \text {where} \nonumber \\ \eta _t^s&= {\left\{ \begin{array}{ll} 1, &  R_t^s = \min (R_t^1,\dots ,R_t^S) \\ 0, &  \text {otherwise} \end{array}\right. }. \end{aligned}$$

By $R_t^s$, we define the loss corresponding to parameter *s*, i.e $R_t^s = \sum _{i=1}^{t-1} \text {Loss}(\hat{y}_t^s,y_t)$. The loss function can be the mean squared error or mean absolute error for general applications. For the application of forecasting future returns, we use the loss function of minus Profit and Loss (abbreviated P&L, and defined in Sect. 4.3).

Similarly, a large *k* for kNN algorithm would produce similar forecasts to using low values of *h*. Instead of using either GRNN or kNN, we use both models, each with their own hyperparameter candidate set, and then select based on performance, in a similar manner to Eq. (36). Aside from these two types of models, we also consider a standard OLS model.

Denote by $S^{\text {GRNN}}$ the hyperparameter candidate for GRNN and $S^{\text {kNN}}$ the hyperparameter candidate set for kNN. Denote by $\hat{y}^{\text {GRNN},s}_t$, the forecast when using GRNN with parameter *s*, $\hat{y}^{\text {kNN},k}_t$ the forecast when using kNN (with parameter *k*), and $\hat{y}_t^{\text {OLS}}$ the forecast when using an OLS model. Then define

37 $$\begin{aligned} \begin{gathered} R^{\text {GRNN},s}_t = \sum _{i=t-L_D}^{t-1} \text {Loss}(\hat{y}^{\text {GRNN},s}_i,y_i). \\ R^{\text {kNN},k}_t = \sum _{i=t-L_D}^{t-1} \text {Loss}(\hat{y}^{\text {kNN},k}_i,y_i). \\ R^{\text {OLS}}_t = \sum _{i=t-L_D}^{t-1} \text {Loss}(\hat{y}^{\text {OLS}}_i,y_i). \end{gathered} \end{aligned}$$

Based on the losses up to time point *t*, the minimum loss is calculated as

38 $$\begin{aligned}  &   R^{\text {optimal}}_t = \text {min}\bigl ( \{ R^{\text {GRNN},s}_t: s \in S^{\text {GRNN}} \} \nonumber \\  &   \cup \{ R^{\text {kNN},k}_t: k \in S^{\text {kNN}} \} \cup \{ R_t^{\text {OLS}} \}\bigr ), \end{aligned}$$

and the number of models achieving the optimal performance as

39 $$\begin{aligned}  &   N^{\text {optimal}}_t = |\{ s: R^{\text {GRNN},s}_t = R^{\text {optimal}}_t \}| \nonumber \\  &   \quad + |\{ k: R^{\text {kNN},k}_t = R^{\text {optimal}}_t\}| + \mathbb {1}({R_t^{\text {OLS}} = R^{\text {optimal}}_t}), \end{aligned}$$

where $\mathbb {1}(A)$ is an indicator function taking the value 1, if condition *A* is satisfied and 0 otherwise. Then we reformulate Eq. (36), to include the kNN and OLS models as

40 $$\begin{aligned} \hat{y}_t =  &   \sum _{s \in S^{\text {GRNN}}} \eta _t^{\text {GRNN},s} \hat{y}^{\text {GRNN},s}_t \nonumber \\  &   + \sum _{k \in S^{\text {kNN}}} \eta _t^{\text {kNN},k} \hat{y}_t^{\text {kNN},k} + \eta _t^{\text {OLS}} \hat{y}_t^{\text {OLS}}, \end{aligned}$$

where

41 $$\begin{aligned} \begin{aligned} \eta ^{\text {GRNN},s}_t = {\left\{ \begin{array}{ll} \frac{1}{ N^{\text {optimal}}_t}, \ \ &  R^{\text {GRNN},s}_t =R^{\text {optimal}}_t \\ 0, \ \ &  \text {otherwise}\\ \end{array}\right. }, \\ \eta ^{\text {kNN},k}_t = {\left\{ \begin{array}{ll} \frac{1}{ N^{\text {optimal}}_t}, \ \ &  R^{\text {kNN},k}_t = R^{\text {optimal}}_t \\ 0, \ \ &  \text {otherwise}\\ \end{array}\right. }, \\ \eta ^{\text {OLS}}_t = {\left\{ \begin{array}{ll} \frac{1}{ N^{\text {optimal}}_t}, \ \ &  R^{\text {OLS}}_t = R^{\text {optimal}}_t \\ 0, \ \ &  \text {otherwise}\\ \end{array}\right. }. \end{aligned} \end{aligned}$$

### Overcoming the shortcomings of kNN and GRNN

One downside of applying dimensionality reduction methods is that they are agnostic of the target to be forecasted. In transforming the initial space in order to retain a large proportion of the variability with a reduced number of features, an initial feature that is already highly correlated with the target but also weakly correlated with other features could end up having a relatively small weight in the final forecasting step. To circumvent this shortcoming, we employ two additional steps.

Firstly, we make sure that all features from the original feature space *X* that have a high enough correlation with the target are retained in the forecasting step. This way, we ensure that selecting the components that capture most of the variability is not done at the expense of dropping original features that provide predictive power on their own. Therefore, we define the following set, after calculating the OSMC between each of the original features and the target

42 $$\begin{aligned} I^{\text {Original}} = \{ j: \text {OSMC}(X_{1:L_0, j },y_{1:L_0}) \ge c_{\text {Original}} \}, \end{aligned}$$

where any feature whose OMSC is higher than the pre-determined $c_{\text {Original}}$, is chosen to be included in the feature set.

During the pre-processing part of the algorithm, we replace $\tilde{X}$ with $(\tilde{X},X_{1:T,I^{\text {Original}}})$, with a number of features equal to $p + |I^{\text {Original}}|$. Note that for mid-sized, low signal-to-error data sets like the daily data set we use in Sect. 4.3, $I_{\text {Original}}$ is usually null.

Secondly, certain features in the embedding space ought to have more influence on the neighbor selection. This can be achieved by using a weighted metric rather than the standard Euclidean norm. In particular, if *v* represents the vector of weights, the weighted Euclidean distance between $X_{t_1}$ and $X_{t_2}$ is given by

43 $$\begin{aligned} || \tilde{X}_{t_1} - \tilde{X}_{t_2} ||_v = \sqrt{\sum _{j=1}^{p + |I^{\text {Original}}|} v_j (\tilde{X}_{t_1,j} - \tilde{X}_{t_2,j})^2}. \end{aligned}$$

To increase robustness we threshold any correlations with absolute values smaller than $c_{\text {min}}$ (which is another hyperparameter) to 0. The weights are based on the OSMC between each feature and the target

44 $$\begin{aligned} v_j = \frac{c_j^2 \mathbb {1}(|c_j | \ge c_{\text {min}})}{\sum \limits _{l=1}^{p + |I^{\text {Original}}|} c_l^2 \mathbb {1}(| c_l | \ge c_{\text {min}})}, \end{aligned}$$

where $$c_j = \text {OSMC}(\tilde{X}_{1:L_0, j },y_{1:L_0}) \ \ \forall j \in \{ 1, \dots , p, p + 1, \dots , |I^\text {Original}| \}$$.

### Algorithm outline

To ensure stationarity of the target *y*, we first perform an ADF test with significance level $p_{\text {ADF}}$, and if the null hypothesis is not rejected, we difference the time series, and repeat until we reject the null hypothesis. We then perform ACF and PACF tests to check if there are any significant ARMA effects.^8^ If such effects are identified, we perform a grid search over the orders of the AR and MA orders (p and q respectively) to find the optimal ARIMA(p,r,q) model in terms of their Bayesian Information Criterion (BIC), where *r* denotes the number of times differencing was applied. Otherwise, we simply fit ARIMA(0,r,0).

Using this procedure, we decompose the target *y* into a time series signal (ARIMA) and a residual component

45 $$\begin{aligned} y = \underbrace{y^{\text {TS}}}_{\text {Time Series Signal}} + \underbrace{\epsilon }_{\text {Residual}}. \end{aligned}$$

Overall, the algorithm consists of three main steps: (1) computing an embedding, (2) choosing features based on the OSMC, and (3) forecasting based on kNN and GRNN. The complete algorithm is given in Algorithm 1, with Algorithm 2 detailing the forecasting step.^9^

**Algorithm 1:**
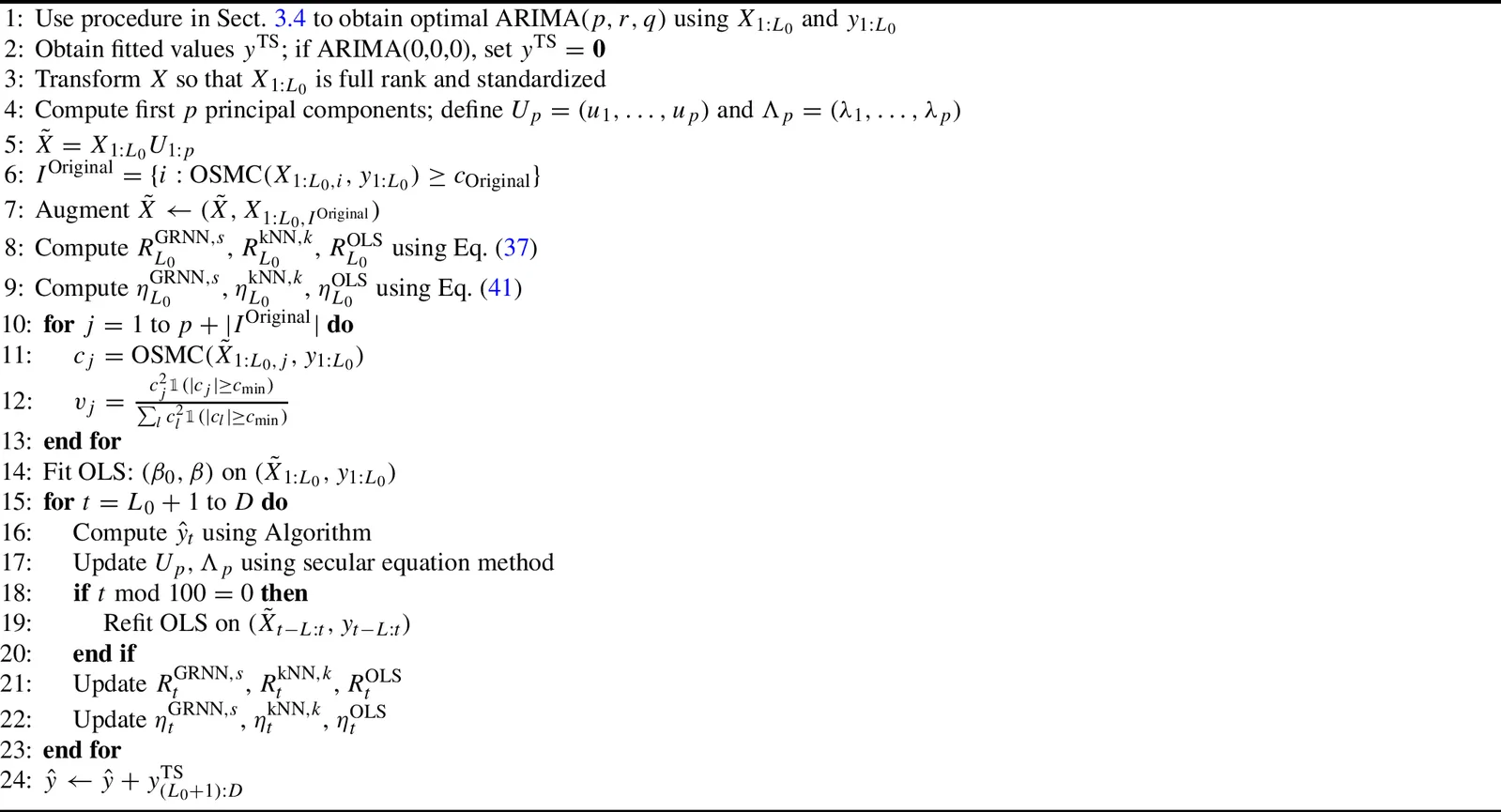
OFTER

**Algorithm 2:**
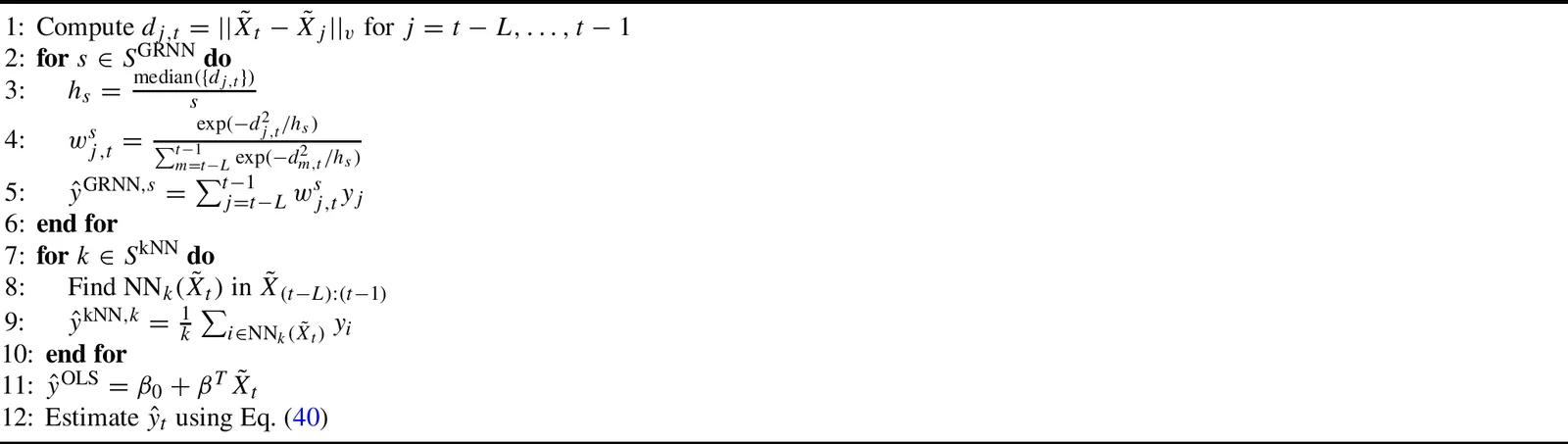
Forecasting for time point *t*

## Empirical results

We consider and compare four different configurations of OFTER, which we denote as follows

- OFTER (no dimensionality reduction and no feature transformation). This entails setting $\tilde{X} = X$, using the Pearson correlation instead of the OSMC, and skipping steps 4, 5, 6, 7, and 17 of Algorithm 1.
- OFTER-DR (dimensionality reduction and no feature transformation). This is almost the same as Algorithm 1, but we use the Pearson correlation instead of the OSMC.
- OFTER-FT (feature transformation and no dimensionality reduction). This entails setting $\tilde{X} = X$ as well as skipping steps 4, 5, 6, 7, and 17 of Algorithm 1.
- OFTER-DR-FT (feature transformation and dimensionality reduction). This is the exact algorithm as given by Algorithm 1.

Unless stated otherwise we use $p_{\text {ADF}} = 0.05$. When performing dimensionality reduction, we consider δ=0.9. For the candidate sets, we use $S^{GRNN} = \{ 0.001,0.005,0.01,$ 0.05,0.1,0.5,1,5,10,50,100} and $S^{\text {kNN}} = \{1,2,3,5,10,$ 15,20,30,50}. In terms of features, we include those corresponding to the current period up to a lag of 3. Lastly, we set $L_0$ equal to $\left\lfloor 0.7 T\right\rfloor$. Note that OFTER is generally robust for different choices of hyperparameters, as evidenced by our robustness analysis reported in Section C.

### Baseline models

We compare the performance of OFTER against that of several mainstream machine learning models. We include the linear models OLS, PCR, LASSO, but also the widely used support vector machines (SVM), random forests, and XGBoost. We also include some of the most widely used time series-tailored deep learning models, such as LSTM, Bi-LSTM, and GRU. Unlike OFTER, most of these methods are not naturally amenable to efficient implementation in an online setting and thus cannot be updated. For comparison purposes, we retrain each of these models every 100 steps. In the case of LSTM, Bi-LSTM, and GRU, we re-fit the model with only five epochs, starting from the previous weight matrix. For the sake of a fair comparison, instead of following the embedding updating step, we also re-compute the embedding of OFTER every 100 steps. We provide an overview of these methods, as well as details of their implementation for this task, in Section B. Note that the features are standardized for all baseline models in the same manner as they are for OFTER.

### Synthetic data sets

Before testing OFTER on real life data sets, we first check whether it can perform consistently well on some simple low dimensional toy models. For this purpose, we use the three five-dimensional models from [25], which are simplistic models crafted to resemble electroencephalography signals and are suitable for this forecasting task.

46 $$\begin{aligned} \text {M1:} {\left\{ \begin{array}{ll} y_t^{(1)} = 0.2 y_{t-1}^{(1)} - 0.4 y_{t-1}^{(2)} + \epsilon _{t}^{(1)} \\ y_t^{(2)} = -0.5 y_{t-1}^{(1)} + 0.15 y_{t-1}^{(2)} + \epsilon _{t}^{(2)} \\ y_t^{(3)} = -0.14 y_{t-1}^{(2)} + \epsilon _{t}^{(3)} \\ y_t^{(4)} = 0.5 y_{t-1}^{(1)} - 0.25 y_{t-1}^{(2)} + \epsilon _{t}^{(4)} \\ y_t^{(5)} = 0.15 y_{t-1}^{(1)} + \epsilon _{t}^{(5)} \end{array}\right. } \end{aligned}$$

The first data set (M1) shown in Eq. (46) is generated by a set of five linear equations.

47 $$\begin{aligned} \mathrm {M2{:}}\quad {\left\{ \begin{array}{ll} y_t^{(1)} = g(y_{t-1}^{(1)}) + \epsilon _{t}^{(1)} \\ y_t^{(2)} = g(y_{t-1}^{(2)}) + 0.5\, y_{t-1}^{(1)} y_{t-1}^{(2)} + \epsilon _{t}^{(2)} \\ y_t^{(3)} = g(y_{t-1}^{(3)}) + 0.3\, y_{t-1}^{(2)} + 0.5(y_{t-1}^{(1)})^2 + \epsilon _{t}^{(3)} \\ y_t^{(4)} = 0.5\, y_{t-1}^{(1)} - 0.25\, y_{t-1}^{(2)} + \epsilon _{t}^{(4)} \\ y_t^{(5)} = 0.15\, y_{t-1}^{(1)} + \epsilon _{t}^{(5)} \end{array}\right. } \end{aligned}$$

where $$g(u) = 3.4u\left( 1-e^{-u^2}\right) e^{-u^2}$$.

The second data set (M2) shown in Eq. (47) is generated by a set of five equations, some of which are non-linear. [25] based these equations on some models appearing in [46].

48 $$\begin{aligned} \text {M3:} {\left\{ \begin{array}{ll} y_t^{(1)} = 0.1 y_{t-1}^{(1)} - 0.6 y_{t-3}^{(2)} + \epsilon _{t}^{(1)} \\ y_t^{(2)} = -0.15 y_{t-3}^{(1)} + 0.8 y_{t-3}^{(2)} + \epsilon _{t}^{(2)} \\ y_t^{(3)} = -0.45 y_{t-3}^{(2)} + \epsilon _{t}^{(3)} \\ y_t^{(4)} = 0.45 y_{t-3}^{(1)} - 0.85 y_{t-3}^{(2)} + \epsilon _{t}^{(4)} \\ y_t^{(5)} = 0.95 y_{t-2}^{(1)} + \epsilon _{t}^{(5)} \end{array}\right. } \end{aligned}$$

The third data set (M3) resembles M1, but is a time series with a higher order, making M3 a VAR model.

In each of the above cases $\epsilon _t^{(1)},\dots ,\epsilon _t^{(5)}$ are uncorrelated normally distributed random variables with unit variance. Note that a burn-in period of 100 periods is used with $y_1^{(1)},\dots ,y_1^{(5)}$ set equal to zero.

### Data

#### ETF data set

We consider a daily data set for two forecasting applications, namely daily returns and volumes. We consider 10 ETFs (SPY, XLF, XLB, XLK, XLV, XLI, XLU, XLY, XLP, XLE). We forecast both daily returns and changes in daily volumes, which we define in the following paragraphs.

Daily Returns: Let $P_{i,d}$ denote the closing price for asset *i* at day *d*. We consider the close to close returns^10^ for asset *i*, defined as

49 $$\begin{aligned} R_{i,d}^{k} = \frac{P_{i,d+k}-P_{i,d}}{P_{i,d}}. \end{aligned}$$

As a more representative quantity, grounded within financial applications, we consider the market excess returns, that is the difference between the returns of an instrument and that of the S&P-500 index, a widely used proxy for market returns.

50 $$\begin{aligned} \tilde{R}_{i,t}^{k} = R_{i,t}^{k} - R_{\text {SPY},t}^{k}. \end{aligned}$$

Predicting daily returns is one of the most popular yet difficult problems in finance. Due to the low signal-to-noise ratio of this task, standard metrics like MSE and correlation, are considered unsuitable. A more suitable economic-driven evaluation method is following a hypothetical transaction of the underlying instruments, based on the forecasted signs (with + and − corresponding to buy and sell, respectively). For each instruments *i* and each day *t*, we denote the forecast as $s_{i,t}$ and execute a hypothetical trade based on the sign of $s_{i,t}$. As this is simply for evaluation purposes, we ignore transaction costs and liquidity constraints.

Following this hypothetical strategy, we consider the (excess-returns-adjusted) P&L metric defined by

51 $$ \begin{aligned} \text {P} \& \text {L}^{k}_d = \frac{1}{N_d} \sum _{i=1}^{N_d} \widetilde{R}^{k}_{i,t} \times \text {sign} \left( s_{i,t} \right) , \end{aligned}$$

The P&L is defined above for each day *d*, which yields a vector ${\textbf {P}} \& {\textbf {L}} = \left( \text {P} \& \text {L}_1,\text {P} \& \text {L}_2,\dots ,\text {P} \& \text {L}_{T-1} \right)$ of daily P&L’s, which measures the outcome of the hypothetical strategy over the entire time horizon under consideration. A measure of the long-term performance of a strategy is the annualized Sharpe ratio (hereafter SR) [47], defined as

52 $$  \begin{aligned} \text {SR} = \frac{\text {mean}({\textbf {P}} \& {\textbf {L}}) \times \sqrt{252}}{\text {sd}({\textbf {P}} \& {\textbf {L}})}. \end{aligned}$$

Lastly, we define the P&L per dollar traded (PPD) as

53 $$ \begin{aligned} \text {PPD} = \text {mean}({\textbf {P}} \& {\textbf {L}}), \end{aligned}$$

which assumes that every day, the total allocated capital to the portfolio remains constant at 1 USD.

We employ the SR significance test by [48]. As returns are known to exhibit heavier tails than a normal distribution, this test is an extension of a traditional *t*-test, adjusted to account for the skewness and kurtosis of the distribution of the returns.

A higher magnitude forecast should naturally signify a higher confidence. For each trading day, we consider the quintiles of the forecast magnitudes $\{|s_{i,t}| \}_{i=1}^N$, in order to define five quintile portfolios, of decreasing size. In particular, we define Q1 to be the portfolio consisting of all assets, Q2 to be the portfolio consisting of the top 80% assets with the highest magnitude, and so on, up to Q5 which consists of the 20% highest magnitude assets.

As naive benchmarks, we follow two naive strategies. The first we call Buy_SPY, which includes buying and holding the SPY ETF. The other, we call Buy_all, which includes buying and holding all of the ETFs for the duration of the test sets.

Daily changes in trading volume: Denote the volume of instrument *i* as $V_{i,d}$, which is defined as the total number of shares traded during day *d*. Note that this quantity is non-stationary and generally increases over time, across most instruments. To this end, we instead consider the ’log-volume-returns’, defined as

54 $$\begin{aligned} \delta ^{k}_{i,d} = \frac{\text {log}(V_{i,d+k})-\text {log}(V_{i,d})}{\text {log}(V_{i,d})}. \end{aligned}$$

Features: The embedding built is common for the two forecasting tasks and is built using a feature matrix consisting of the following

- 1, 2 and 3-day returns for each of the instruments, i.e $R^1_{i,d}$, $R^2_{i,d}$, $R^3_{i,d}$
- 1, 2 and 3-day absolute returns for each of the instruments, i.e $|R^1_{i,d}|$, $|R^2_{i,d}|$, $|R^3_{i,d}|$
- 1, 2 and 3-day log-volume returns for each of the instruments, i.e $\delta ^1_{i,d}, \delta ^2_{i,d},\delta ^3_{i,d}$
- the three Fama & French daily market factors
- the daily returns of 49 different industry portfolios^11^
- daily returns of the VIX index

### Evaluation metrics

To evaluate the forecasting models in this section we make use of the following evaluation metrics. Denote the vector of predicted values $\hat{y}$ and the observed vector *y*.

**Root Mean Squared Error (RMSE)**:

55 $$\begin{aligned} \text {RMSE} = \sqrt{\frac{1}{T} \sum _{t=1}^T \left( y_{t} - \hat{y}_{t}\right) ^2}. \end{aligned}$$

**Mean Absolute Error (MAE)**:

56 $$\begin{aligned} \text {MAE} = \frac{1}{T} \sum _{t=1}^T \left| y_{t} - \hat{y}_{t} \right| . \end{aligned}$$

**Spearman Correlation**: The Spearman Correlation between $\hat{y}_t$ and *y* is the Pearson correlation between the ranks of the two vectors. This allows this metric to capture nonlinear monotonic relations. The Pearson correlation between $\hat{y}$ and *y* is given by

57 $$\begin{aligned} C = \frac{\sum _{t=1}^T (y_t - \bar{y}) (\hat{y}_{t} - \bar{\hat{y}})}{\sqrt{\sum _{t=1}^T (y_t - \bar{y})^2 (\hat{y}_{t} - \bar{\hat{y}}}{})^2}. \end{aligned}$$

In addition, we use the **Diebold-Mariano (DM) test** to compare two different sets of forecasts, which we denote by $\{ \hat{y}_t \}_{t=1}^T$ (for the model considered) and $\{ n_t \}_{t=1}^T$ (for the naive forecasts). When *y* corresponds to a change (such as the daily return, or change in volumes) the naive forecast is set to $n_t = 0$. Otherwise, the naive forecast is set to $n_t = y_{t-1}$. The DM test is a popular variant of the paired t-test introduced in [50]. When using loss function *g*, the difference in the loss of the two forecasts at time point *t* is given by $d_t = g(n_t - y_t) - g(\hat{y}_t - y_t)$. The one-tailed version of DM that we use tests whether the considered forecasts $\{ \hat{y}_t \}_{t=1}^T$ outperform the naive forecasts $\{ n_t \}_{t=1}^T$. In particular it tests the null hypothesis

58 $$\begin{aligned} H_0: \mathbb {E} \left( d_t \right) = 0 \end{aligned}$$

versus the alternative hypothesis

59 $$\begin{aligned} H_1: \mathbb {E} \left( d_t \right) \ge 0. \end{aligned}$$

In our case, we use the squared error loss, i.e $g(e) = e^2$, and a significance level of 5%.

Lastly, we use an instance of the Theil-U statistic (TU) [51]. As pointed out by [52], it is recommended to use the second version of this statistic, which is defined as

60 $$\begin{aligned} U = \sqrt{\frac{\sum _t \left( y_t - \hat{y}_t \right) ^2}{\sum _t \left( y_t - n_t\right) ^2}}. \end{aligned}$$

**Table 1:** For each forecasted quantity in each of the three multivariate time series the running time for each of the 12 mentioned methods is recorded

Data Set	T	d	LM	PCR	LASSO	Random Forests	SVM	LSTM	Bi-LSTM	GRU	OFTER	OFTER-DR	OFTER-FT	OFTER-DR-FT
Synthetic	3000	15	0.01	0.02	0.64	33.90	1.81	173.45	202.99	184.81	7.87	9.68	37.52	24.53
Financial	5022	154	0.37	0.48	5.38	3,144.02	0.17	442.86	503.78	549.30	17.58	22.25	222.87	153.06
Air Quality	1461	228	0.10	0.31	2.23	132.05	1.00	165.48	152.22	184.85	3.25	20.18	157.95	62.64

The average running time (in seconds) of each of the 12 methods across the different forecasted quantities is given, where *T* denotes the length of the time series, and *d* denotes the dimensionality of each data set. Note that the Air Quality dataset mentioned here is explored in Section E

### Numerical results

We begin with a discussion of the running time of OFTER. In Table 1, we provide the average running time of the algorithm for each of the three considered data sets. We notice that all four configurations of OFTER are significantly faster than LSTM, Bi-LSTM, and GRU, while at the same time significantly slower than the OLS, LASSO, PCR, and SVM, as expected. It is also clear that among the OFTER configurations, OFTER-FT is the slowest as each feature is transformed separately without first reducing the dimensionality of the feature space. The time complexity of OFTER and OFTER-DR are more comparable, with the latter being slightly more computationally intensive.

For the numerical experiments concerning forecasting returns, we employ economic-driven metrics typical of this literature, as discussed in Sect. 4.3. For all the remaining experiments, we base the performance comparisons on RMSE and the other metrics defined in Sect. 4.4.

#### Synthetic data

**Fig. 3:**
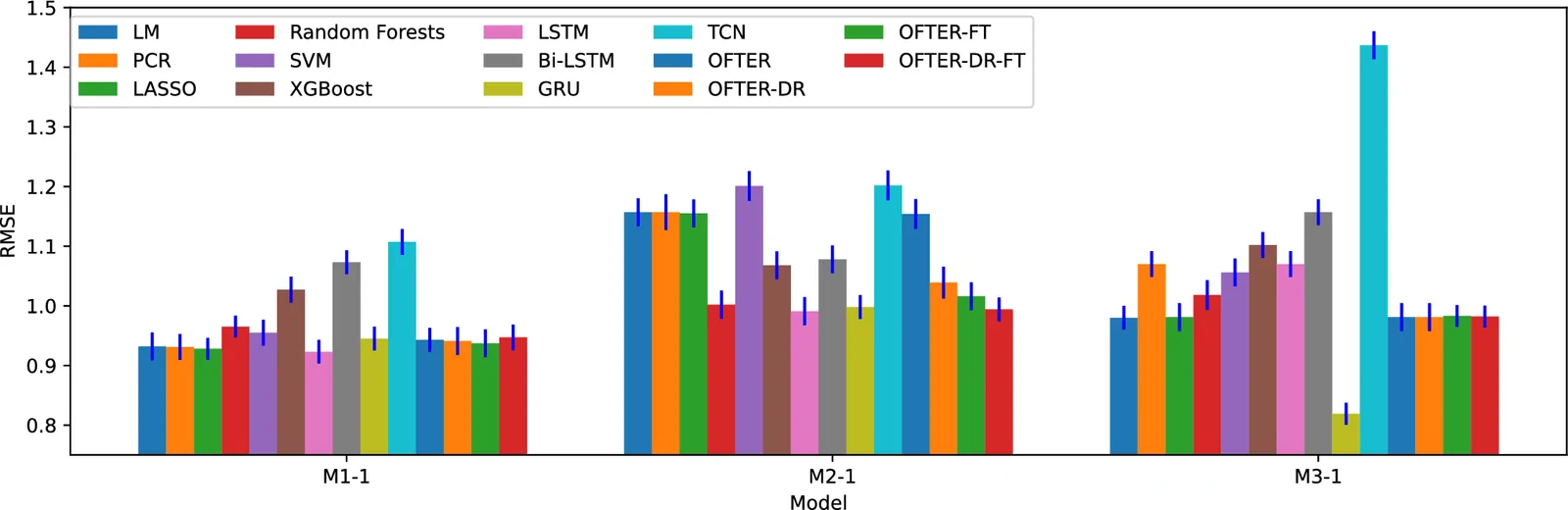
Grouped barplot showing a performance comparison of all methods for the synthetic data set. The metric of the comparison is the RMSE between the target values and the forecasts. The bootstrapped standard errors are also displayed

For this task, we use $L = 800, c_{\text {min}} = 0.05$ and $c_{\text {Original}} = 0.05, L_D = 500$. The results are given in Table 2 with the RMSE scores for M1-1, M2-1 and M3-1 visualized in Fig. 3. Importantly, in most cases OFTER has a *p*-value of zero for the DM test, and TU statistics that are significantly lower than 1. Two exceptions are M1.3 (which is structurally a very low signal-to-ratio model), and M2.1 for which the OFTER configurations that do not use the feature transformation component score very poorly (similar to other linear models).

For M1, the best-performing models are generally the linear models LM, PCR, and LASSO which correspond to linear models. GRU and LSTM also outperform the OFTER configurations in a number of cases, with differences being small in most cases. On the other hand, OFTER generally outperforms random forests, XGBoost and SVM. In the case of M2, which is a nonlinear multivariate model, the configurations of OFTER that employ the feature transformation component generally perform the best, with the exception of random forests, LSTM, and GRU. In this case, OFTER outperforms the linear models as well as XGBoost. Lastly, in the case of M3, which regards a vector autoregressive (VAR) model, OFTER is performing particularly well, outperforming all other models, with the exception of GRU, and having similar performances to the LM and LASSO models.

**Table 2:** In this table, we summarize the performance of 14 models for each of the three synthetic models described in Sect. 4.2

M1	M1.1				M1.2				M1.3				M1.4				M1.5			
	MSE	MAE	TU	DM	MSE	MAE	TU	DM	MSE	MAE	TU	DM	MSE	MAE	TU	DM	MSE	MAE	TU	DM
LM	0.932	0.745	0.882	0.000	0.978	0.774	0.860	0.000	1.017	0.820	0.992	0.031	0.965	0.756	0.824	0.000	0.978	0.777	0.983	0.000
PCR	0.931	0.744	0.880	0.000	0.979	0.774	0.861	0.000	1.015	0.820	0.991	0.012	0.969	0.763	0.827	0.000	0.977	0.777	0.982	0.000
LASSO	0.928	0.740	0.877	0.000	0.977	0.772	0.859	0.000	1.014	0.818	0.989	0.027	0.965	0.759	0.824	0.000	0.976	0.776	0.981	0.000
Random Forests	0.965	0.766	0.913	0.000	0.979	0.781	0.860	0.000	1.034	0.832	1.009	0.200	0.972	0.765	0.829	0.000	0.990	0.784	0.995	0.010
SVM	0.955	0.764	0.903	0.000	1.029	0.812	0.904	0.000	1.061	0.854	1.035	0.131	1.013	0.804	0.865	0.000	1.001	0.796	1.006	0.029
XGBOOST	1.027	0.820	0.972	0.000	1.073	0.856	0.943	0.000	1.114	0.893	1.086	0.480	1.079	0.859	0.921	0.000	1.087	0.871	1.093	0.123
LSTM	0.923	0.728	1.000	0.091	0.946	0.755	0.982	0.002	1.010	0.821	1.001	0.866	0.975	0.772	1.003	0.135	0.980	0.787	0.998	0.039
Bi-LSTM	1.073	0.859	1.163	0.605	1.054	0.851	1.093	0.967	1.141	0.913	1.131	0.599	1.094	0.883	1.125	0.003	1.084	0.860	1.105	0.346
GRU	0.945	0.748	1.024	0.012	0.933	0.747	0.968	0.000	1.012	0.826	1.003	0.683	0.938	0.742	0.965	0.000	1.002	0.804	1.020	0.416
TCN	1.107	0.892	0.980	0.000	1.163	0.921	0.963	0.000	1.009	0.805	0.996	0.000	1.158	0.921	0.969	0.000	1.021	0.816	1.000	0.000
OFTER	0.943	0.753	0.892	0.000	0.990	0.786	0.869	0.000	1.024	0.828	0.998	0.300	0.971	0.763	0.829	0.000	0.986	0.783	0.991	0.002
OFTER-DR	0.941	0.750	0.890	0.000	0.987	0.782	0.867	0.000	1.019	0.826	0.995	0.060	0.970	0.762	0.828	0.000	0.980	0.779	0.985	0.003
OFTER-FT	0.937	0.747	0.886	0.000	0.981	0.779	0.861	0.000	1.020	0.825	0.995	0.073	0.965	0.756	0.824	0.000	0.977	0.774	0.982	0.000
OFTER-DR-FT	0.947	0.756	0.896	0.000	0.984	0.781	0.864	0.000	1.020	0.826	0.995	0.021	0.963	0.760	0.822	0.000	0.979	0.777	0.984	0.001

M2	M2.1				M2.2				M2.3				M2.4				M2.5			
	MSE	MAE	TU	DM	MSE	MAE	TU	DM	MSE	MAE	TU	DM	MSE	MAE	TU	DM	MSE	MAE	TU	DM
LM	1.157	0.918	1.004	0.590	1.374	1.113	0.999	0.261	1.520	1.141	0.879	0.000	1.025	0.822	0.815	0.000	1.036	0.827	0.991	0.001
PCR	1.157	0.917	1.004	0.542	1.376	1.113	1.001	0.248	1.553	1.172	0.898	0.000	1.052	0.843	0.837	0.000	1.036	0.827	0.991	0.001
LASSO	1.155	0.915	1.003	0.500	1.373	1.112	0.998	0.485	1.519	1.140	0.879	0.000	1.025	0.821	0.815	0.000	1.033	0.825	0.988	0.000
Random Forests	1.002	0.802	0.869	0.000	1.040	0.832	0.757	0.000	1.066	0.832	0.617	0.000	1.067	0.860	0.848	0.000	1.043	0.837	0.998	0.001
SVM	1.201	0.949	1.042	0.741	1.249	1.005	0.908	0.000	1.253	0.985	0.725	0.000	1.066	0.863	0.848	0.000	1.069	0.849	1.023	0.002
XGBOOST	1.068	0.853	0.927	0.000	1.153	0.920	0.840	0.000	1.125	0.886	0.651	0.000	1.152	0.920	0.916	0.000	1.125	0.904	1.076	0.069
LSTM	0.991	0.787	1.002	0.285	0.990	0.805	1.002	0.072	1.029	0.781	1.006	0.215	1.030	0.820	1.002	0.470	1.040	0.838	1.000	0.356
Bi-LSTM	1.078	0.864	1.090	0.601	1.104	0.877	1.116	0.323	1.130	0.921	1.104	0.170	1.130	0.903	1.098	0.277	1.145	0.921	1.100	0.108
GRU	0.998	0.793	1.010	0.568	1.002	0.814	1.013	0.011	1.021	0.778	0.998	0.010	1.013	0.808	0.985	0.000	1.048	0.842	1.008	0.932
TCN	1.202	0.957	1.000	0.000	1.463	1.151	0.997	0.000	1.541	1.199	0.946	0.000	1.236	0.984	0.993	0.000	1.022	0.825	1.001	0.000
OFTER	0.981	0.790	0.542	0.000	0.965	0.763	0.422	0.000	1.012	0.811	0.697	0.000	0.995	0.797	0.364	0.000	0.998	0.804	0.495	0.000
OFTER-DR	0.981	0.791	0.542	0.000	0.961	0.760	0.420	0.000	1.009	0.807	0.694	0.000	0.995	0.800	0.364	0.000	0.995	0.803	0.494	0.000
OFTER-FT	0.983	0.791	0.543	0.000	0.972	0.769	0.425	0.000	1.008	0.804	0.694	0.000	0.992	0.802	0.363	0.000	0.995	0.800	0.493	0.000
OFTER-DR-FT	0.982	0.791	0.542	0.000	0.979	0.778	0.428	0.000	1.014	0.809	0.698	0.000	1.001	0.806	0.366	0.000	0.999	0.802	0.495	0.000

M3	M3.1				M3.2				M3.3				M3.4				M3.5			
	MSE	MAE	TU	DM	MSE	MAE	TU	DM	MSE	MAE	TU	DM	MSE	MAE	TU	DM	MSE	MAE	TU	DM
LM	0.980	0.787	0.541	0.000	0.965	0.765	0.422	0.000	1.005	0.807	0.692	0.000	1.000	0.801	0.366	0.000	0.996	0.799	0.494	0.000
PCR	1.070	0.851	0.591	0.044	1.081	0.854	0.473	0.000	1.077	0.860	0.742	0.900	1.204	0.955	0.440	0.018	1.195	0.968	0.593	0.000
LASSO	0.981	0.787	0.542	0.000	0.965	0.764	0.422	0.000	1.004	0.806	0.691	0.000	1.001	0.803	0.366	0.000	0.995	0.799	0.494	0.000
Random Forests	1.018	0.820	0.562	0.000	1.021	0.806	0.446	0.000	1.038	0.826	0.714	0.000	1.041	0.833	0.381	0.000	1.031	0.833	0.512	0.000
SVM	1.056	0.840	0.583	0.000	1.108	0.874	0.484	0.000	1.056	0.843	0.727	0.000	1.148	0.888	0.420	0.000	1.076	0.856	0.534	0.000
XGBOOST	1.102	0.883	0.608	0.000	1.123	0.893	0.491	0.000	1.119	0.894	0.769	0.000	1.140	0.909	0.417	0.000	1.097	0.890	0.545	0.000
LSTM	1.070	0.852	1.021	0.538	1.080	0.859	1.026	0.404	0.978	0.787	0.932	0.202	1.105	0.889	1.050	0.602	1.087	0.875	1.019	0.004
Bi-LSTM	1.157	0.933	1.104	0.981	1.146	0.918	1.089	0.040	1.166	0.942	1.112	0.642	1.128	0.912	1.072	0.049	1.157	0.946	1.084	0.000
GRU	0.819	0.648	0.781	0.000	0.899	0.714	0.854	0.000	1.034	0.828	0.985	0.363	0.881	0.698	0.837	0.198	0.906	0.723	0.849	0.035
TCN	1.437	1.138	0.850	0.475	1.679	1.327	0.883	0.000	1.208	0.972	0.885	0.000	1.810	1.433	0.796	1.778	1.512	1.173	0.820	0.000
OFTER	0.981	0.790	0.542	0.000	0.965	0.763	0.422	0.000	1.012	0.811	0.697	0.000	0.995	0.797	0.364	0.000	0.998	0.804	0.495	0.000
OFTER-DR	0.981	0.791	0.542	0.000	0.961	0.760	0.420	0.000	1.009	0.807	0.694	0.000	0.995	0.800	0.364	0.000	0.995	0.803	0.494	0.000
OFTER-FT	0.983	0.791	0.543	0.000	0.972	0.769	0.425	0.000	1.008	0.804	0.694	0.000	0.992	0.802	0.363	0.000	0.995	0.800	0.493	0.000
OFTER-DR-FT	0.982	0.791	0.542	0.000	0.979	0.778	0.428	0.000	1.014	0.809	0.698	0.000	1.001	0.806	0.366	0.000	0.999	0.802	0.495	0.000

**Table 3:** In this table, we summarize the performance of 14 models for the delta volume forecasting task for each of the ETFs

	SPY				XLF				XLB				XLK				XLV			
	MSE	MAE	TU	DM	MSE	MAE	TU	DM	MSE	MAE	TU	DM	MSE	MAE	TU	DM	MSE	MAE	TU	DM
LM	**0**.**016**	0.013	0.860	0.000	0.020	0.015	0.938	0.000	0.025	0.020	0.947	0.000	**0**.**022**	0.017	0.881	0.000	0.023	0.019	0.967	0.000
PCR	**0**.**016**	0.013	0.861	0.000	0.019	**0**.**014**	0.883	0.000	0.024	0.019	0.903	0.000	**0**.**022**	0.017	0.857	0.000	**0**.**022**	0.018	0.920	0.000
LASSO	**0**.**016**	0.013	0.864	0.000	**0**.**018**	**0**.**014**	0.867	0.000	**0**.**023**	**0**.**018**	0.886	0.000	**0**.**022**	0.017	0.859	0.000	**0**.**022**	**0**.**017**	0.909	0.000
Random Forests	**0**.**016**	0.013	0.865	0.000	0.019	**0**.**014**	0.875	0.000	**0**.**023**	**0**.**018**	**0**.**860**	0.000	**0**.**022**	0.017	0.864	0.000	**0**.**022**	**0**.**017**	0.908	0.000
SVM	0.019	0.015	1.003	0.010	0.021	0.017	0.997	0.000	0.030	0.023	1.124	0.000	0.030	0.025	1.176	0.000	0.033	0.026	1.354	0.000
XGBOOST	0.017	0.014	0.915	0.000	0.021	0.016	0.969	0.000	0.026	0.020	0.973	0.000	0.024	0.019	0.963	0.000	0.026	0.020	1.058	0.000
LSTM	0.980	0.774	0.996	0.007	0.509	0.396	1.001	0.255	0.452	0.355	1.009	0.023	0.629	0.487	1.000	0.011	0.372	0.291	1.018	0.024
Bi-LSTM	1.100	0.882	1.117	0.669	0.704	0.586	1.384	0.008	0.665	0.560	1.483	0.078	0.797	0.653	1.267	0.069	0.632	0.550	1.729	0.835
GRU	0.962	0.760	0.978	0.000	0.520	0.409	1.023	0.000	0.470	0.364	1.050	0.014	0.640	0.497	1.018	0.528	0.391	0.308	1.069	0.016
TCN	0.045	0.034	2.089	0.000	0.100	0.078	4.665	0.000	0.059	0.046	2.154	0.000	0.105	0.080	4.075	0.000	0.083	0.063	3.390	0.000
OFTER	**0**.**016**	0.013	0.862	0.000	0.019	0.015	0.883	0.000	**0**.**023**	**0**.**018**	0.893	0.000	**0**.**022**	0.017	0.883	0.000	**0**.**022**	**0**.**017**	0.915	0.000
OFTER-DR	**0**.**016**	**0**.**012**	**0**.**844**	0.000	**0**.**018**	**0**.**014**	0.860	0.000	**0**.**023**	**0**.**018**	0.886	0.000	**0**.**022**	**0**.**016**	**0**.**851**	0.000	**0**.**022**	**0**.**017**	0.905	0.000
OFTER-FT	**0**.**016**	**0**.**012**	0.853	0.000	**0**.**018**	**0**.**014**	0.862	0.000	**0**.**023**	**0**.**018**	0.881	0.000	**0**.**022**	0.017	0.859	0.000	**0**.**022**	**0**.**017**	**0**.**888**	0.000
OFTER-DR-FT	**0**.**016**	**0**.**012**	0.856	0.000	**0**.**018**	**0**.**014**	**0**.**858**	0.000	**0**.**023**	**0**.**018**	0.888	0.000	**0**.**022**	0.017	0.860	0.000	**0**.**022**	**0**.**017**	0.915	0.000

	XLI				XLU				XLY				XLP				XLE			
	MSE	MAE	TU	DM	MSE	MAE	TU	DM	MSE	MAE	TU	DM	MSE	MAE	TU	DM	MSE	MAE	TU	DM
LM	0.025	0.019	1.046	0.000	0.021	0.016	0.958	0.000	0.025	0.020	0.956	0.000	0.023	0.018	0.902	0.000	0.020	0.015	0.993	0.000
PCR	0.022	**0**.**017**	0.937	0.000	0.020	0.015	0.902	0.000	0.023	**0**.**018**	0.901	0.000	**0**.**022**	0.018	0.866	0.000	0.018	0.015	0.925	0.000
LASSO	**0**.**021**	**0**.**017**	0.906	0.000	**0**.**019**	**0**.**014**	**0**.**862**	0.000	0.023	**0**.**018**	0.889	0.000	**0**.**022**	**0**.**017**	0.857	0.000	0.018	0.014	0.898	0.000
Random Forests	**0**.**021**	**0**.**017**	0.912	0.000	**0**.**019**	0.015	0.876	0.000	**0**.**022**	**0**.**018**	0.868	0.000	**0**.**022**	**0**.**017**	**0**.**856**	0.000	**0**.**017**	0.014	0.881	0.000
SVM	0.030	0.024	1.278	0.000	0.030	0.024	1.379	0.000	0.033	0.026	1.260	0.000	0.028	0.022	1.085	0.000	0.027	0.022	1.397	0.000
XGBOOST	0.026	0.020	1.115	0.000	0.022	0.018	1.039	0.000	0.027	0.021	1.032	0.000	0.026	0.020	1.004	0.000	0.020	0.016	1.012	0.000
LSTM	0.310	0.243	1.015	0.987	0.389	0.303	1.025	0.166	0.360	0.279	1.041	0.874	0.469	0.353	0.999	0.001	0.404	0.318	1.031	0.081
Bi-LSTM	0.604	0.537	1.976	0.844	0.628	0.543	1.652	0.686	0.616	0.539	1.779	0.168	0.687	0.585	1.462	0.001	0.639	0.550	1.632	0.797
GRU	0.323	0.255	1.057	0.000	0.406	0.319	1.068	0.058	0.370	0.288	1.068	0.002	0.498	0.381	1.060	0.288	0.410	0.325	1.048	0.003
TCN	0.077	0.059	3.241	0.000	0.062	0.047	2.752	0.000	0.070	0.054	2.500	0.000	0.052	0.040	1.783	0.024	0.056	0.044	2.829	0.000
OFTER	0.022	**0**.**017**	0.935	0.000	**0**.**019**	0.015	0.887	0.000	0.023	**0**.**018**	0.886	0.000	0.023	0.018	0.901	0.000	0.018	0.014	0.898	0.000
OFTER-DR	0.022	**0**.**017**	0.929	0.000	**0**.**019**	**0**.**014**	0.863	0.000	0.023	**0**.**018**	0.880	0.000	0.023	0.018	0.888	0.000	**0**.**017**	**0**.**013**	**0**.**876**	0.000
OFTER-FT	**0**.**021**	**0**.**017**	**0**.**904**	0.000	**0**.**019**	**0**.**014**	0.868	0.000	**0**.**022**	**0**.**018**	**0**.**858**	0.000	**0**.**022**	**0**.**017**	0.859	0.000	**0**.**017**	0.014	0.880	0.000
OFTER-DR-FT	0.022	**0**.**017**	0.932	0.000	**0**.**019**	**0**.**014**	0.871	0.000	**0**.**022**	**0**.**018**	0.866	0.000	0.023	0.018	0.885	0.000	**0**.**017**	0.014	**0**.**876**	0.000

#### ETF volumes

For both financial tasks, we use L=800 and set $c_{\text {min}} = c_{\text {Original}} = 0.01$ and $L_D = 300$, as typically, these are problems with a lower signal-to-noise ratio. For the task of predicting the delta-volumes of ETF instruments Eq. (54), OFTER outperforms all other benchmark methods. As demonstrated in Table 3, the best performing model is an OFTER configuration in seven out of ten cases, with random forests being the best performing for XLB and XLP, and LASSO being the best performing for XLU. In addition, in all cases, for all OFTER configurations, the *p*-value corresponding to the DM test is equal to zero up to four decimal places.

#### ETF market excess returns

**Table 4:** In this table, we summarize the performance of 14 models for the ETF price movement forecasting task

	Q1					Q2					Q3					Q4					Q5				
	SR	SR (costs)	PPD	PPD (costs)	*p*-value	SR	SR (costs)	PPD	PPD (costs)	*p*-value	SR	SR (costs)	PPD	PPD (costs)	*p*-value	SR	SR (costs)	PPD	PPD (costs)	*p*-value	SR	SR (costs)	PPD	PPD (costs)	*p*-value
LM	0.275	−0.148	1.300	−0.700	0.540	0.204	−0.202	1.005	−0.995	0.650	0.301	−0.068	1.631	−0.369	0.502	0.380	0.026	2.148	0.148	0.395	0.476	0.165	3.066	1.066	0.287
PCR	0.063	−0.355	0.301	−1.699	0.889	0.011	−0.385	0.055	−1.945	0.980	0.003	−0.362	0.015	−1.985	0.995	0.018	−0.329	0.102	−1.898	0.968	0.116	−0.193	0.748	−1.252	0.796
LASSO	−0.149	−0.571	−0.706	−2.706	0.739	−0.164	−0.566	−0.819	−2.819	0.713	−0.158	−0.524	−0.866	−2.866	0.723	−0.180	−0.529	−1.037	−3.037	0.687	−0.495	−0.780	−3.462	−5.462	0.270
Random Forests	0.309	−0.202	1.209	−0.791	0.489	0.349	−0.140	1.428	−0.572	0.435	0.325	−0.103	1.519	−0.481	0.468	0.305	−0.092	1.536	−0.464	0.495	0.460	0.141	2.887	0.887	0.303
SVM	0.288	−0.149	1.317	−0.683	0.520	0.161	−0.260	0.766	−1.234	0.719	0.286	−0.085	1.540	−0.460	0.524	0.232	−0.121	1.315	−0.685	0.604	−0.025	−0.332	−0.160	−2.160	0.956
XGBoost	−0.333	−1.114	−0.852	−2.852	0.458	−0.298	−0.996	−0.853	−2.853	0.507	−0.403	−0.992	−1.369	−3.369	0.371	−0.347	−0.884	−1.289	−3.289	0.442	−0.237	−0.624	−1.224	−3.224	0.598
LSTM	0.266	−0.606	0.609	−1.386	0.551	0.523	−0.281	1.297	−0.698	0.238	−0.078	−0.753	−0.230	−2.225	0.862	−0.193	−0.776	−0.661	−2.656	0.666	0.443	0.048	2.236	0.241	0.321
Bi-LSTM	−0.951	−1.298	−5.476	−7.471	0.036	−0.868	−1.214	−4.993	−6.989	0.056	−0.867	−1.205	−5.121	−7.116	0.056	−0.921	−1.255	−5.516	−7.512	0.042	−0.982	−1.294	−6.269	−8.264	0.031
GRU	0.212	−0.599	0.522	−1.473	0.633	0.239	−0.517	0.631	−1.365	0.590	0.121	−0.506	0.386	−1.610	0.786	0.050	−0.516	0.177	−1.819	0.911	−0.006	−0.417	−0.030	−2.025	0.989
TCN	0.212	−0.599	0.522	−1.473	0.633	0.239	−0.517	0.631	−1.365	0.590	0.121	−0.506	0.386	−1.610	0.786	0.050	−0.516	0.177	−1.819	0.911	−0.006	−0.417	−0.030	−2.025	0.989
OFTER	1.163	0.576	3.967	1.967	0.009	1.103	0.548	3.970	1.970	0.014	1.126	0.633	4.564	2.564	0.012	1.099	0.640	4.782	2.782	0.015	0.479	0.129	2.737	0.737	0.285
OFTER-DR	1.310	0.730	4.515	2.515	0.003	1.235	0.683	4.477	2.477	0.005	0.894	0.415	3.730	1.730	0.041	0.677	0.215	2.929	0.929	0.128	0.669	0.301	3.634	1.634	0.136
OFTER-FT	1.143	0.559	3.915	1.915	0.011	1.151	0.610	4.254	2.254	0.011	0.947	0.466	3.938	1.938	0.035	0.769	0.314	3.383	1.383	0.087	0.461	0.111	2.634	0.634	0.302
OFTER-DR-FT	1.163	0.576	3.967	1.967	0.009	1.103	0.548	3.970	1.970	0.014	1.126	0.633	4.564	2.564	0.012	1.099	0.640	4.782	2.782	0.015	0.479	0.129	2.737	0.737	0.285

Here, *p*-value denotes the *p*-value obtained from the one-tailed SR significance test of [48]. Q1-Q5 correspond to the quantile portfolios as defined in Sect. 4.3. Costs include a 2 basis point round-trip trading cost

**Fig. 4:**
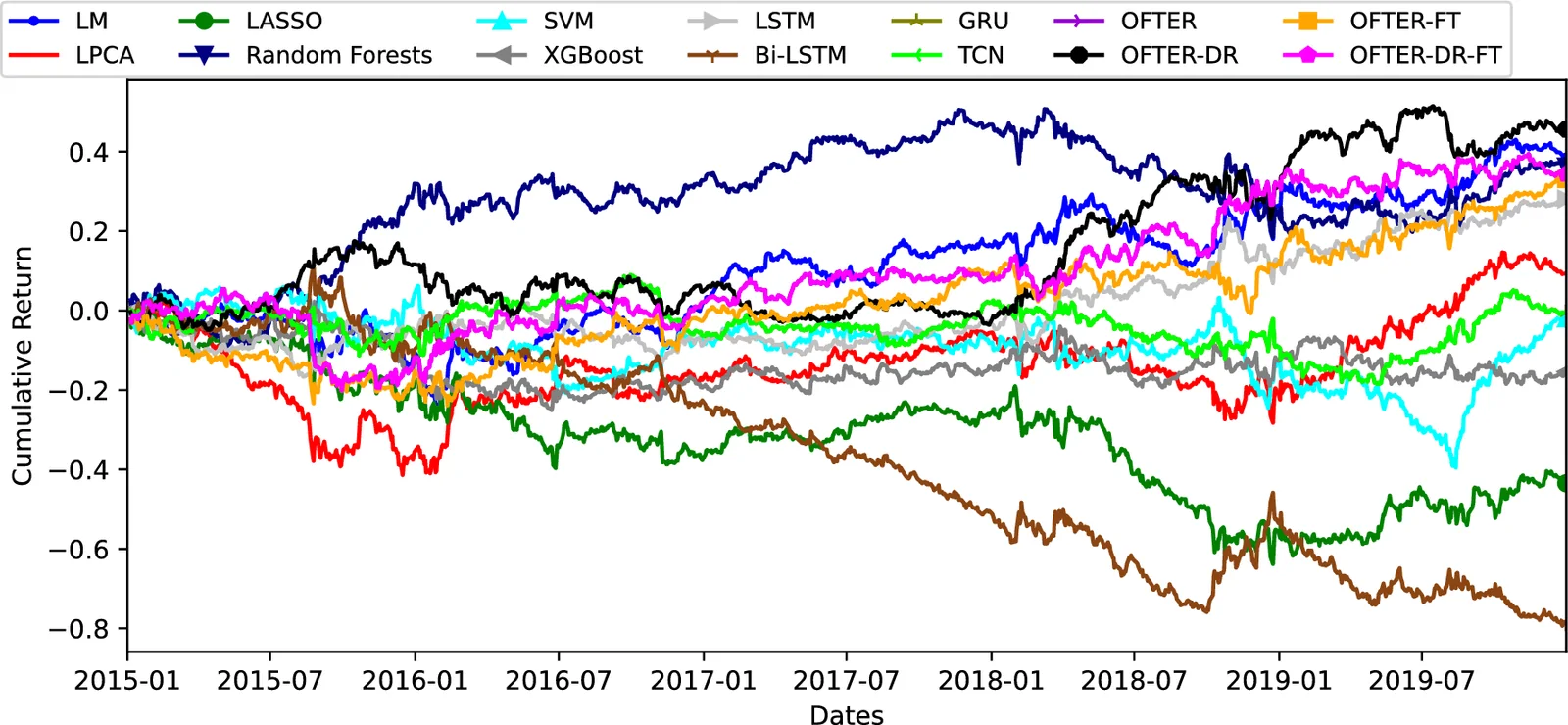
Cumulative P&L plot of the daily strategies corresponding to the 14 methods for the ETF price movement forecasting task. Here, *p*-value denotes the *p*-value as obtained from the one-tailed Sharpe Ratio significance test of [48]. The performance metrics for each method are given in Table 4

We simulate a daily strategy based on trading ten ETF instruments for the Q1-Q5 portfolios, and summarize the results in Table 4. For three out of four OFTER strategies, the SR is considered statistically significant.^12^ As a visual demonstration of the results, we also plot the cumulative P&L for the Q1 strategy of each method in Fig. 4, clearly demonstrating that OFTER-DR and OFTER-DR-FT are outperforming the other methods. With the exception of the Q4-Q5 Bi-LSTM strategy, none of the other benchmark methods result in strategies with a statistically significant SR. Across Q1 and Q2, OFTER-DR scores the highest performance, both in terms of SR and PPD. In Fig. 4, we plot the cumulative P&L for the OFTER-DR strategy, clearly demonstrating a consistently well-performing set of strategies across different quantiles.

An application to an air pollution data set is given in Section E.

## Applications to feature importance and outlier detection

In this section, we discuss additional applications of OFTER to two tasks of interest in the machine learning community. In particular, we consider a feature importance component that boosts the interpretative power of OFTER, and an application to outlier detection.

### Feature importance

**Fig. 5:**
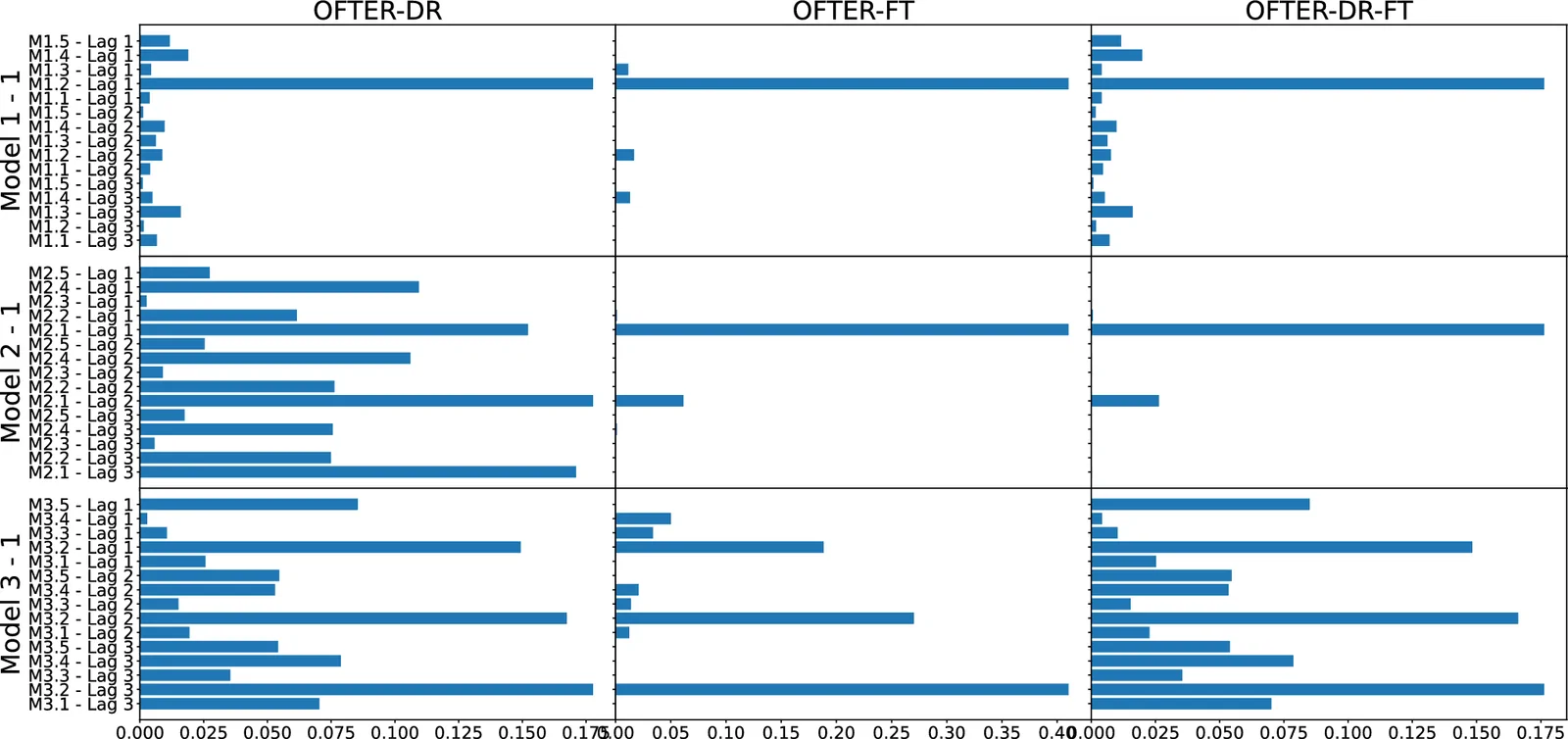
In this 3x3 plot grid, we show the feature importance weights, as obtained in Sect. 5.1. The first feature of each of M1, M2, and M3 is forecasted in the first, second, and third row, respectively. In each case, there are 15 features corresponding to three lags of each of the five features. This is repeated for three of OFTER’s configurations, corresponding to the three columns of the grid

In the OFTER pipeline, one can put forth a natural approach for estimating the contribution of each feature to the forecasting task. In particular, we aim to find a proxy for the importance of each of the input features. To this end, consider two points in the initial feature space $x_1$ and $x_2$, and denote by $x_1' = U^T x_1$ and $x_2' = U^T x_2$ the transformed variables. Denote by $D' = || x_1' - x_2' ||_v$ the distance in the reduced space (used for calculating the nearest neighbors), and let $D_j = x_{1,j}- x_{2,j}$ such that $||x_1 - x_2||_2 = \sqrt{\sum _j D_j^2}$.

A metric for the importance of a variable in determining the relative distance of data points in the embedding space can be modeled as the sensitivity of the distance $D^{\prime }$ on the individual distance $$d_j$$, i.e $$\left| \frac{\partial D'}{\partial D_j}\right| .$$ For our goal, it is natural to take the derivative at a point where $$D_1 = \dots = D_d = 1$$. We begin by writing

61 $$\begin{aligned} D'^2&= \Vert x_1' - x_2' \Vert _{v} = \Vert U^T (x_1 - x_2) \Vert _{v} \nonumber \\&= \sum _{j=1}^p v_j \left( \sum _{k=1}^d U_{k,j} (x_1-x_2)_k \right) ^2 \nonumber \\&= \sum _{j=1}^p v_j \left( \sum _{k=1}^d U_{k,j} D_k \right) ^2. \end{aligned}$$

We then compute

62 $$\begin{aligned} \frac{\partial D'^2}{\partial D_k}\Bigr |_{d = e_d} = 2 \sum _{j=1}^p v_j \left( \sum _{k'=1}^d U_{k',j} D_{k'} \right) U_{k,j}, \end{aligned}$$

which, using ⊙ to denote the Hadamard product, can be written as

63 $$\begin{aligned} \nabla D'^2(e_d) = 2 \left| U \left[ (U^T e_d) \odot v \right] \right| . \end{aligned}$$

Keeping in mind Eq. (42), we also augment the embedding space with the original features that are highly correlated with the target. Consider the vector $v_2$ containing the weight given to the original feature if used, and zero otherwise, i.eThis now leads to

64 $$\begin{aligned} D'^2 = \sum _{j=1}^p v_j \left( \sum _{k=1}^d U_{k,j} D_k \right) ^2 + \sum _{l=1}^d v_{2,l} D_l^2, \end{aligned}$$

and thus the derivative is instead given by

65 $$\begin{aligned} \nabla D'^2(e_d) = 2 \left| U \left[ (U^T e_d) \odot v \right] + v_2\right| . \end{aligned}$$

As an illustration, we consider the task of forecasting the first feature of each of the three synthetic data sets (M1-M3), using OFTER-DR, OFTER-FT, and OFTER-DR-FT. In Fig. 5, we show a grid of the feature importance of each individual feature. As we ignore the unimportant features via the calculation of *v* in Eq. (44), OFTER-FT attaches a non-zero importance weight only to a subset of the features. On the other hand, OFTER-DR assigns a non-zero weight to all of the original features, as they all contribute to the estimation of each principal component. This also holds true for OFTER-DR-FT, where dimensionality reduction is also performed. However, in the case of M2 where most of the predictability is present in a single feature (M2.1 - Lag1), OFTER-DR-FT assigns a zero importance weight to most of the features, similarly to OFTER-FT. Therefore, for the task of feature importance, we recommend the user to rely on either OFTER–FT or OFTER-DR-FT.

### Outlier detection

**Fig. 6:**
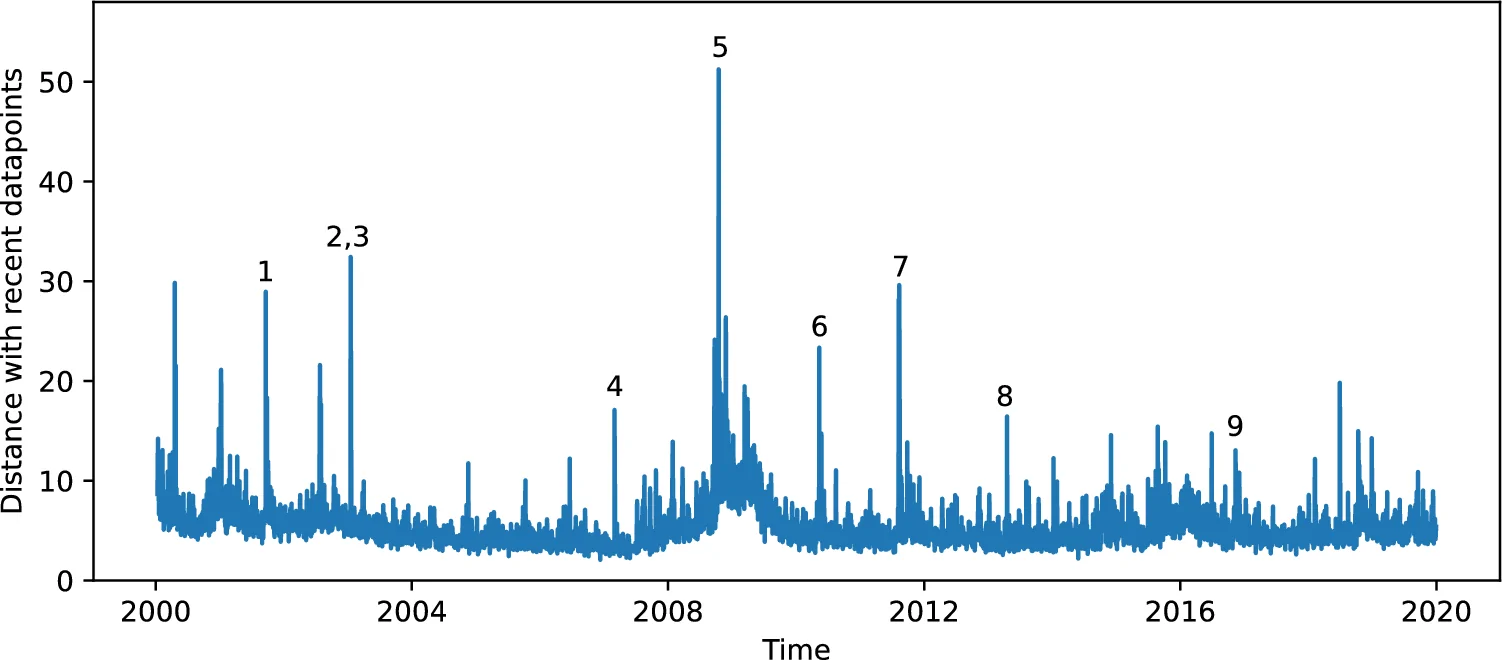
Plot showing the minimum distance $d_j^\text {min}$ obtained using the suggested outlier detection algorithm for OFTER-DR, L=600 and κ=5. All numbered points are identified as outliers. 1. 18 September 2001 - Aftermath of the 9/11 Attack 2. 24 July 2002 - Stock market downturn of 2002 ($12^\text {th}$ consecutive day of falling prices) 3. 29 July 2002 - Stock market downturn of 2002 ($3^\text {rd}$ day of rising prices after sharp price rises following (2)) 4. 28 February 2007 - Big drop in US and China stocks 5. 10 October 2008 - Market Crash across Europe and Asia 6. 7 May 2010 - Following the Flash Crash of May 6 7. 4 August 2011 - Preceding the Black Monday (8 August) 8. 15 April 2013 - One of the biggest drops in 2013 for DJIA, SPX 9. 8 November 2016 - US election

A byproduct of the OFTER pipeline is that it can facilitate the task of outlier detection, by considering the pairwise distance between time points in the embedding space. More specifically, points whose nearest neighbors are still quite “far" in the embedding space could be labeled as outliers, in the same spirit as the one-class SVM algorithm [53]. We demonstrate this using the following example.

Define the minimum distance between the current time point *t* and the time points in its recent history in the reduced space as

66 $$\begin{aligned} d_{t}^\text {min} = \text {min}(\{ d_{t-L,t}, \dots , d_{t-1,t} \}). \end{aligned}$$

Comparing $d_{t}^\text {min}$ with the distribution of $(d_{t-L}^\text {min},\dots ,d_{t-1}^\text {min})$ may constitute input for any outlier detection algorithm.^13^ Denoting by $q^{\text {min}}_{t,1}$ and $q^{\text {min}}_{t,3}$ the first and third quantiles of $\{ d^{\text {min}}_{t-L}, \dots , d^{\text {min}}_{t-1} \}$, we classify a time point *t* as an outlier if $d_{j}^\text {min} > q^{\text {min}}_{t,3} + \kappa \left( q^{\text {min}}_{t,3} - q^{\text {min}}_{t,1} \right)$ for some κ>0.

In Fig. 6, we plot the minimum distance between each trading day and its L=600 preceding days, by using κ=5. From this figure, one could infer several different regimes, as there are periods where the levels of $d_\text {min}$ are low, and periods where these are high, such as the periods around the 2008 financial crisis. The fact that almost all labeled outliers fall in the immediate vicinity of days significant in the financial history, does indeed indicate that the algorithm can successfully be used to uncover abnormality signals.

Finally, we remark that selecting a larger value of *L* would result in a large number of anomalies being highlighted. In practice, if this outlier detection approach is used in a trading application, a smaller κ would be desirable, as the outlier warning could possibly be qualitatively analyzed. Note that the above quantile-based outlier detection approach is just a sample algorithm to illustrate the potential of OFTER, as the pipeline is amenable to be incorporated into any other outlier detection algorithm.

## Conclusion and future work

In this paper, we have introduced OFTER, a complete pipeline for online multivariate time series forecasting. By leveraging kNN and GRNN, both simple well-understood methods with trivial training complexities, OFTER manages to outperform a number of baselines, for mid-sized data sets. In particular, an extensive set of numerical experiments demonstrated that OFTER performed competitively with random forests and GRU in the synthetic data set, and outperformed all other methods in the real-world applications considered, with a running time much lower than those of deep learning methods. The shortcomings due to the curse of dimensionality are overcome via dimensionality reduction and the use of a weighted norm when comparing distances in the embedding space.

Furthermore, OFTER brings additional advantages, including its flexible nature, with the ability to use or omit many of its components. As a byproduct, OFTER can be integrated seamlessly into an outlier detection pipeline, in an online manner. Lastly, OFTER is naturally equipped to return the user a feature importance score for all the input variables.

**Future work.** Future extensions of this work could include the utilization of other machine learning methods in the model selection step of the algorithm, for example, LASSO models with different regularization parameters. One disadvantage of OFTER is that it struggles to discover nonlinear interaction effects between covariates, even though this is a general issue with models and data sets of this size. One potential extension would be to include a pre-screening component, where a number of potential interaction terms are tested in order to augment the feature space, either prior to or after the embedding step. Additionally, it would be interesting to see the performance of OFTER in other mid-sized data sets and assess whether other dimensionality reduction algorithms are able to attain improved performance, relative to the use of PCA (this was not the case for the data sets used in this project). Yet another interesting direction concerns an adaptation of OFTER for probabilistic forecasting. Probabilistic models have recently gained in popularity, being able to provide not only forecasts but also associated uncertainty estimates, which is of crucial importance in certain applications. In addition, multi-step probabilistic forecasting is another area which could provide a natural extension for the pipeline presented in this paper. For the financial applications, we evaluate primarily the predictive quality of the generated signals rather than fully implementable trading performance. While we report results incorporating a simple transaction-cost adjustment, we do not account for other important trading frictions such as price slippage and market impact. Incorporating these effects is an important direction for future work. Lastly, we note that the OSMC metric we introduced is of independent interest, both as an alternative to the maximal correlation but also as a computationally fast additional step for other simple regression methods.

## Figures and Tables

**Fig. 7:**
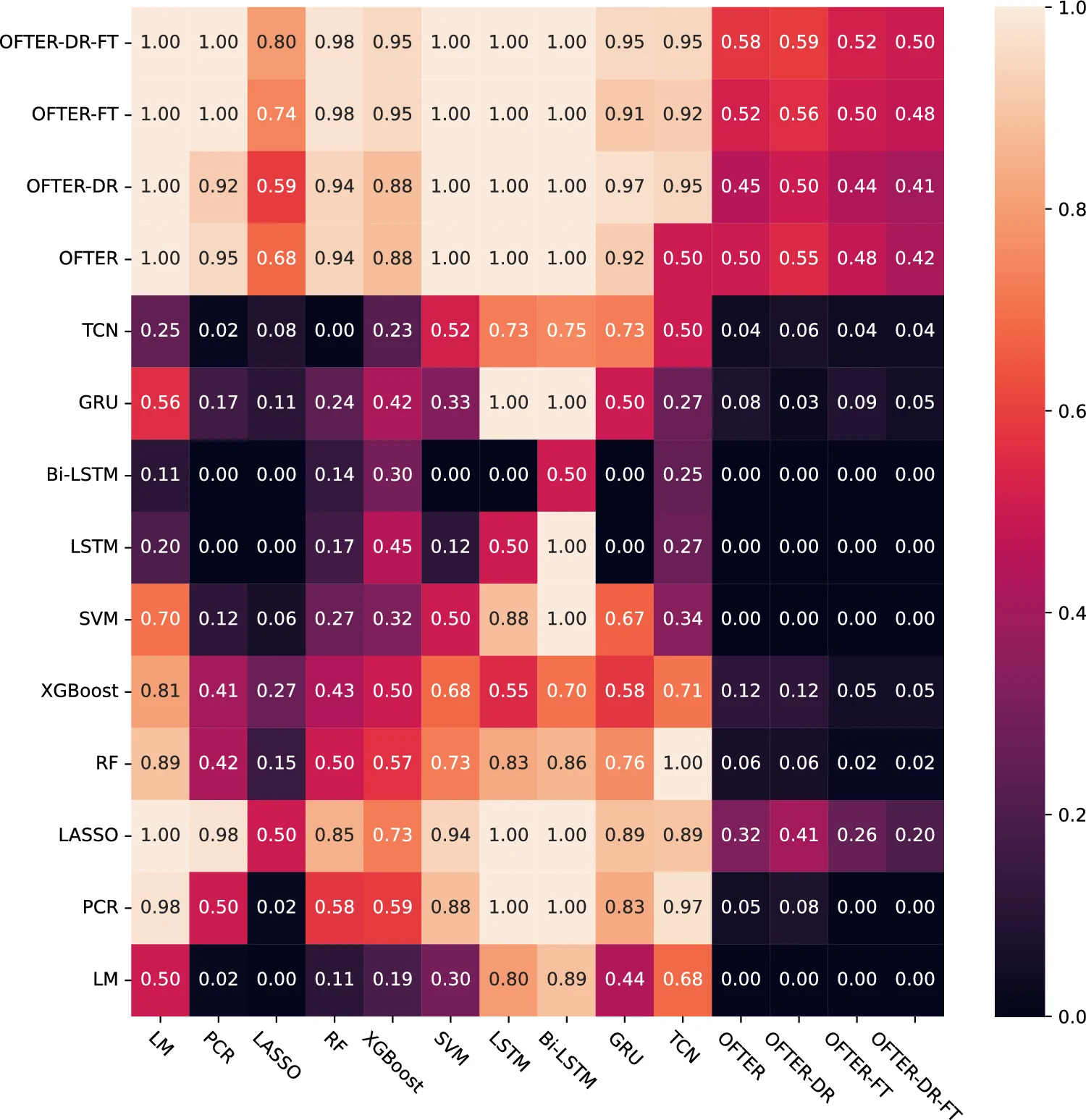
This heatmap is a visualization of the comparison between the different forecasting models for the air pollution data set. The proportion of the number of cases where a method (label of the vertical axis) outperforms another method (label of the horizontal axis) in terms of RMSE given in the corresponding cell

## Data Availability

No datasets were generated or analyzed during the current study.

## Appendix A: A convexifying approach for solving the secular equation

In this section, we give a brief description of the method we use to solve the secular equation described in Sect. 3.1, which amounts to finding the roots of

A1 $$\begin{aligned} f(\kappa ) = 1 + \rho \sum _{j=1}^d \frac{z_j^2}{\lambda _j-\kappa }. \end{aligned}$$

The main difficulty in directly solving Eq. (A1) lies in the multitude of discontinuities in the domain of *f*. An efficient approach to find the $i^{\text {th}}$ root of the equation, would be to consider the reparameterizations $\kappa = \lambda _i + \rho \xi$ and $\delta _j = \frac{\lambda _j - \lambda _i}{\rho }$, which leads to the formulation of Eq. (A1) as

A2 $$\begin{aligned} f_i(\xi ) = 1 + \sum _{j=1}^d \frac{z_j^2}{\delta _j - \xi }. \end{aligned}$$

By assuming that each eigenvalue is distinct (a reasonable assumption when dealing with real data), we notice that

A3 $$\begin{aligned} \delta _1< \delta _2< \dots< \delta _i = 0< \dots < \delta _n. \end{aligned}$$

Therefore, we look for solutions in the set $\left( 0, \delta _{i+1} \right)$, for i<n, and in $\mathbb {R}_+$ for i=n. We can further transform $f_i(x)$ into a convex function, by considering $F_i(\gamma ) = f_i(\gamma ^{-1/2})$, which can be shown to transform $F_i$ into a convex function in the interval $(\frac{1}{\delta _{i+1}},\infty )$ for i<n and (0,∞) for i=n.

Finally, by using the fact that, for a decreasing convex function, the Newton method^14^ monotonically converges to a solution [54], it can be established that, if $\gamma ^*$ is the solution to $F_i(\gamma ) = 0$, then the Newton method converges monotonically to $\gamma ^*$ with any starting point in $\left[ \frac{1}{\delta _{i+1}^2},\gamma ^* \right]$. For a more detailed explanation and an alternative based on the secant method, we refer the reader to [42].

## Appendix B: Brief summary of baseline methods

The following methods are used as baselines against OFTER. We provide a short description of each one.

### Ordinary least squares (OLS)

Ordinary least squares (OLS) is by far the most well-known regression method, where the target variable *y* is modeled as a linear combination of the features

B4 $$\begin{aligned} y_i = \beta _0 + \sum _{j=1}^d \beta _j X_{i,j} + \epsilon _{i}. \end{aligned}$$

The coefficient vector $\beta = (\beta _0,\dots ,\beta _d)$, is chosen as the one maximizing $\sum \epsilon _{i}^2$, and can be found using the closed-form formula

B5 $$\begin{aligned} \beta = (X^T X)^{-1} X^T y. \end{aligned}$$

Since we include lagged features in the feature matrix, this method is very similar to a Vector Autoregressive Regression (VAR) model.

### Principal component regression

To combat the high correlation present in the features, Principal Component Regression (PCR) first identifies the principal components of *X*, obtaining $\tilde{X}$, and then a number of components p<d is retained. OLS is then used, but on the first *p* columns of $\tilde{X}$ instead of *X*. Similar to OFTER, we use δ=0.9 for choosing the number of components.

### Least absolute shrinkage and selection operator

An alternative to reducing the dimensionality of *X* before OLS, is to include a regularization term in the objective function Eq. (B4). Least Absolute Shrinkage and Selection Operator (LASSO) is a widely popular method, where the modified objective function takes the following form

B6 $$\begin{aligned} (y - X \beta )^T (y - X \beta ) + \lambda \sum _j | \beta _j |. \end{aligned}$$

Note that in this case, there is no closed-form solution, and thus a numerical optimization method is utilized. We choose λ using a cross-validation procedure.

### Random forests

A decision tree is a simple method inspired by decision analysis, where the visualization of the model resembles a tree consisting of nodes and branches. The initial feature space constitutes the root node. The observations in the root node are partitioned into ’successor nodes’, based on a splitting rule chosen so as to optimize some criterion (usually either entropy or Gini Impurity). The process is repeated recursively on each of the successor nodes until some stopping criterion is met. For each new data point, its features are then used to determine its corresponding leaf node. Forecasts can then be obtained by averaging the target value of all observations that fall within each leaf node. This is a non-parametric method, but with a very high variance.

A Random Forest is built by fitting multiple decision trees. Each decision tree is fitted using only a random sample of observations and a random sample of features. The forecasts of the decision trees are then combined into a single forecast (usually by averaging). Random Forests are based on the idea of bagging aimed at reducing variance and avoiding overfitting.

For the benchmark task, we use 200 trees, which are built using the squared error criterion. The proportion of used features is set to 0.333, as recommended by [55].

**Table 5:** Robustness analysis of OFTER. The table displays the correlation between the target and forecasts for each of the 15 features of the synthetic data set, by varying a subset of five parameters of the algorithm

	δ		$p_{\text {ADF}}$		*L*		$C_{\text {min}}$		$C_{\text {original}}$	
Value	0.8	0.95	0.01	0.1	500	1500	0.01	0.1	0.01	0.1
M1.1	0.470	0.459	0.467	0.467	0.441	0.477	0.464	0.464	0.465	0.461
M1.2	0.519	0.511	0.509	0.509	0.503	0.521	0.509	0.514	0.504	0.518
M1.3	0.077	0.071	0.085	0.085	0.085	0.113	0.119	0.116	0.085	0.117
M1.4	0.554	0.558	0.552	0.552	0.555	0.556	0.553	0.556	0.545	0.559
M1.5	0.094	0.107	0.101	0.101	0.147	0.120	0.102	0.110	0.101	0.113
M2.1	0.599	0.623	0.599	0.599	0.596	0.603	0.675	0.600	0.599	0.60
M2.2	0.535	0.535	0.535	0.535	0.534	0.539	0.897	0.535	0.536	0.536
M2.3	0.864	0.799	0.815	0.815	0.776	0.850	0.759	0.401	0.804	0.817
M2.4	0.962	0.964	0.963	0.963	0.963	0.963	0.962	0.963	0.963	0.963
M2.5	0.679	0.681	0.681	0.681	0.678	0.682	0.681	0.678	0.680	0.679
M3.1	0.758	0.758	0.758	0.758	0.751	0.762	0.758	0.758	0.758	0.757
M3.2	0.852	0.851	0.851	0.851	0.85	0.852	0.851	0.850	0.849	0.850
M3.3	0.669	0.664	0.665	0.665	0.662	0.668	0.665	0.666	0.664	0.667
M3.4	0.902	0.903	0.903	0.903	0.902	0.905	0.903	0.902	0.903	0.902
M3.5	0.826	0.827	0.827	0.827	0.825	0.828	0.827	0.826	0.825	0.825

### XGBoost

Boosting is a class of ensemble models, where the weak learners usually take the form of decision trees. At each iteration, a new weak learner is fitted whose training phase focuses on the previously misclassified data points. The eXtreme Gradient Boosting (XGBoost) is one of the most widely used variants of boosting. We use the standard sklearn implementation of XGBoost, with the default parameters.

### Support vector machines

SVM is another popular machine learning model that is motivated by learning theory. In particular, a vector β and a value $\beta _0$ are found that minimize

B7 $$\begin{aligned} \frac{1}{2} ||\beta ||^2, \ \ \ \text {s.t} \ \ \ | (y_i - \beta ^T x_i - \beta _0) | \le \epsilon . \end{aligned}$$

A prediction for a new point $x^{\prime }$ is then given by $$\beta _0 + \beta ^T x'$$. We use the default settings of sklearn, by choosing ϵ=0.1.

### Recurrent neural networks

Recurrent neural networks (RNN) form a class of neural networks specifically aimed at data sets with temporal structures. This is generally achieved by introducing additional skip connections that pass the output of a past layer to the current one. One issue caused is that gradients have the tendency to vanish or explode.

LSTM is a variant of RNN, specifically designed to avoid the issue of exploding or vanishing gradients. This is achieved by introducing a cell state, which contains a summary of the previous cells and is controlled by a number of different gates. Denote by $x_t$ the feature vector of a data point, $c_t$ the input of the cell state, and $h_t$ the output of the module. The formula of the basic LSTM takes the form

B8 $$\begin{aligned} {\left\{ \begin{array}{ll} f_t = \sigma \left( W_f x_t + U_f h_{t-1} + b_f \right) , \\ i_t = \sigma \left( W_i x_t + U_i h_{t-1} + b_i \right) , \\ o_t = \sigma \left( W_o x_t + U_o h_{t-1} + b_o \right) , \\ \hat{c}_t = \text {tanh} \left( W_c x_t + U_c h_{t-1} + b_c \right) , \\ c_t = f_t \odot c_{t-1} + i_t \odot \hat{c}_t. h_t = c_t \odot \text {tanh}(c_t), \\ \end{array}\right. } \end{aligned}$$

where $W_f,U_f,b_f,W_i,U_i,b_i,W_o,U_o,b_o,W_c,U_c$ and $b_c$ are trainable parameters.

We consider two more variants of LSTM. Bi-LSTM which utilizes two parallel and reverse networks of LSTMs so that information can flow in both directions. We also use Gated Recurrent Unit (GRU) which has a similar yet simpler architecture than LSTM.

For each variant, the layout consists of two layers, each with a unit size of 20, and a fully connected layer. The lookback period is set equal to 3, to match the data used for the other methods. For the training, 50 epochs appear to be enough. For optimization ADAM is used with learning parameter equal to 0.0001, and the other parameters set as default in the corresponding Keras package.

**Table 6:** Sensitivity of results to ARIMA order selection

		Fixed Order					
Task	Metric	(0,0,0)	(1,0,0)	(0,0,1)	(2,0,0)	(1,0,1)	BIC-Selected
M1.1	MSE	0.912	0.915	0.914	0.927	0.924	0.928
M2.1	MSE	1.092	0.991	1.000	0.997	0.997	1.000
M3.1	MSE	1.043	1.045	1.066	1.072	1.060	1.060
SPX ETF Ret	SR	0.431	−0.389	0.375	0.033	0.371	0.371
SPX ETF Vol	MSE	0.0217	0.0201	0.0192	0.0195	0.0189	0.0189

### Temporal convolutional networks

Temporal Convolutional Networks (TCNs) are a class of convolutional neural networks specifically designed and widely used for time series modeling. They rely on one-dimensional dilated causal convolutions, which enforce the temporal structure, and allow the receptive field of the network to grow rapidly without a corresponding increase in the number of parameters.

The TCN architecture we employ consists of three causal convolutional layers with 32 filters and dilation rates of 1, 2, and 4, respectively, each using a kernel size of 2 and ReLU activation. The convolutional layers are followed by a global average pooling layer and a fully connected output layer. The model is trained for 100 epochs using the ADAM optimizer with a learning rate of 0.0001. The lookback period is set equal to 3.

## Appendix C: Robustness analysis

In this short section, we demonstrate how changing five of the main parameters used in OFTER-DR-FT affects its performance on the synthetic data set. We fix five of the main parameters given in OFTER as follows: δ=0.9, L=800, $c_\text {min} = 0.05$, $c_\text {Original} = 0.05$, and $p_{\text {ADF}} = 0.05$. By keeping the other four parameters fixed, we vary each of these parameters in turn and note the variation in its performance. In Table 5, we show the correlation between the target and the forecasts when changing each of the noted parameters to the given value.

In general, the performance of the algorithm does not significantly depend on the choice of parameters, as forecasting each feature has consistent performance. However, there are exceptions; for example, $c_\text {min}$ appears to have a significant effect on the performance of the algorithm for M2.3 and M2.4.

## Appendix D: Effect of ARIMA order

In Step 1 of the OFTER pipeline, the ARIMA order is selected via BIC. To assess sensitivity to this choice, we fix the ARIMA order exogenously and re-run the forecasting experiments for a representative subset of tasks. The results are reported in Table 6. The findings are broadly stable across specifications. For the synthetic tasks (M1.1, M2.1, M3.1), MSE varies only marginally across orders, with differences typically in the second or third decimal place. For the ETF return task, the Sharpe ratio fluctuates somewhat more, which is expected given the noisier nature of return forecasting—notably, ARIMA(0,0,0) performs comparably to the BIC-selected order. For the volume forecasting task, results are again stable. Overall, the choice of ARIMA order has limited impact on the conclusions.

## Appendix E: Non-financial application

As a non-financial application we also consider the Beijing air quality data set from the UCI Machine Learning Repository. Data are collected by the Beijing Municipal Environmental Monitoring Center, and considers various air quality measurements from 12 monitoring points. We aim at forecasting each of the six types of pollution (PM2.5,PM10,03,C0,S02,N02) concentrations (in $ug/m^3$), for each of the stations. Other features in the data set, used as predictors, include Temperature, Dewpoint Temperature, Precipitation, Pressure as well as Wind speed and Direction.

We use L=800 and set $c_\text {min} = c_\text {Original} = 0.01, L_D = 500$ In Fig. 7, we show a grid of the proportions of cases where one method outperforms another based on the RMSE metric. As it can be seen, all four OFTER configurations are outperforming all of the other ten benchmarks. In this particular case, OFTER-DR-FT outperforms the other three versions of OFTER.

## Appendix F: Theoretical results for one-sided maximal correlation

Let $(x_i, y_i)_{i=1}^{N}$ be i.i.d. copies of a pair (*x*, *y*) with x,y∈[0,1] almost surely. Let $\phi _1, \ldots , \phi _K: [0,1] \rightarrow \mathbb {R}$ be a fixed finite collection of measurable feature functions, and define the feature matrix $\boldsymbol{\Phi }\in \mathbb {R}^{N \times K}$ by $\boldsymbol{\Phi }_{i,j} = \phi _j(x_i)$. Write $\textbf{y} = (y_1,\ldots ,y_N)^\top$, $\bar{y} = \frac{1}{N}\sum _{i=1}^N y_i$, and $\textbf{e}_N = (1,\ldots ,1)^\top \in \mathbb {R}^N$. The objects of interest are

F9 $$\begin{aligned} A \;=\; \frac{\boldsymbol{\Phi }^\top \boldsymbol{\Phi }}{N-1}, \qquad \textbf{z} \;=\; \boldsymbol{\Phi }^\top \textbf{y}, \qquad \bar{\textbf{z}} \;=\; \boldsymbol{\Phi }^\top (\textbf{y} - \bar{y}\,\textbf{e}_N), \end{aligned}$$

and the coefficient vector

F10 $$\begin{aligned} c_N \;=\; \frac{A^{-1}\bar{\textbf{z}}}{\sqrt{\bar{\textbf{z}}^\top A^{-1} \textbf{z}}}. \end{aligned}$$

Their population counterparts are the K×K Gram matrix *G* and vectors $\textbf{g}, \boldsymbol{\mu }_z \in \mathbb {R}^K$ with entries

$$\begin{aligned}&G_{jk} \;=\; \mathbb {E}[\phi _j(x)\phi _k(x)], \qquad g_j \;=\; \textrm{Cov}(\phi _j(x), y), \qquad \\&\mu _{z,j} \;=\; \mathbb {E}[\phi _j(x)\,y]. \end{aligned}$$

### Assumption 1

(i.i.d. bounded observations) $(x_1,y_1),\ldots ,$ $(x_N,y_N)$ are i.i.d. with $x_i, y_i \in [0,1]$ almost surely.

### Assumption 2

(Bounded feature functions) There exists C<∞ such that $|\phi _j(v)| \le C$ for all v∈[0,1] and all j=1,…,K.

### Assumption 3

(Invertibility) The population Gram matrix *G* is invertible.

Assumptions 1 and 2 together ensure all products $\phi _j(x_i)\phi _k$ $(x_i)$ and $\phi _j(x_i)y_i$ are bounded and square-integrable. Assumption 3 holds whenever $\phi _1,\ldots ,\phi _K$ are linearly independent in their function space, i.e. no nontrivial linear combination vanishes almost everywhere under the distribution of *x*.

### Lemma 1

(Convergence of the empirical Gram matrix) Under Assumptions 1–2, $A \xrightarrow {\,p\,}G$ in the Frobenius norm as $N\rightarrow \infty$.

### Proof

The (*j*, *k*) entry of *A* is $\frac{1}{N-1}\sum _{i=1}^N \phi _j(x_i)\phi _k(x_i)$, a sample mean of i.i.d. bounded random variables with population mean $G_{jk}$. Entry-wise convergence in probability to $G_{jk}$ follows from the Weak Law of Large Numbers (WLLN) and the N/(N-1)→1 correction via Slutsky’s theorem. Since *K* is fixed, this implies Frobenius convergence. □

### Lemma 2

(Convergence of the centered cross-moment vector) Under Assumptions 1–2, $\bar{\textbf{z}}/(N-1) \xrightarrow {\,p\,}\textbf{g}$ as $N\rightarrow \infty$.

### Proof

The *j*-th entry of $\bar{\textbf{z}}/(N-1)$ decomposes as

$$\begin{aligned}  &   \frac{1}{N-1}\sum _{i=1}^N \phi _j(x_i)(y_i - \bar{y}) \;=\; \frac{1}{N-1}\sum _{i=1}^N \phi _j(x_i)(y_i - \mu _y) \nonumber \\  &   \;-\; (\bar{y} - \mu _y)\cdot \frac{1}{N-1}\sum _{i=1}^N \phi _j(x_i), \end{aligned}$$

where $\mu _y = \mathbb {E}[y]$. The first term converges in probability to $g_j$ by the WLLN, the summands being i.i.d. and bounded by 2*C*. The second term vanishes in probability since $\bar{y} - \mu _y \xrightarrow {\,p\,}0$ by the WLLN and $\frac{1}{N-1}\sum _i \phi _j(x_i)$ is bounded in probability. Slutsky’s theorem gives the result entry-wise, and vector convergence follows since *K* is fixed. □

### Lemma 3

(Convergence of the uncentred cross-moment vector) Under Assumptions 1–2, $\textbf{z}/(N-1) \xrightarrow {\,p\,}\boldsymbol{\mu }_z$ as $N\rightarrow \infty$.

### Proof

The proof is identical to that of Lemma 2 without the vanishing centering term. □

### Theorem 4

(Consistency of $c_N$) Under Assumptions 1–3, with N>K and $\textbf{g}^\top G^{-1}\boldsymbol{\mu }_z > 0$,

$$ c_N \;\xrightarrow {\,p\,}\; \textrm{C}^* \;\equiv \; \frac{G^{-1}\textbf{g}}{\sqrt{\textbf{g}^\top G^{-1}\boldsymbol{\mu }_z}} \qquad \text {as } N \rightarrow \infty . $$

### Proof

Setting $\tilde{\textbf{z}} = \bar{\textbf{z}}/(N-1)$ and $\tilde{\textbf{u}} = \textbf{z}/(N-1)$, we have $c_N = A^{-1}\tilde{\textbf{z}} / \sqrt{\tilde{\textbf{z}}^\top A^{-1}\tilde{\textbf{u}}}$. Lemmas 1–3 give joint convergence $(A, \tilde{\textbf{z}}, \tilde{\textbf{u}}) \xrightarrow {\,p\,}(G, \textbf{g}, \boldsymbol{\mu }_z)$. The continuous mapping theorem applied to $M \mapsto M^{-1}$, $(M,\textbf{v})\mapsto M\textbf{v}$, and $(M,\textbf{v},\textbf{u}) \mapsto \sqrt{\textbf{v}^\top M\textbf{u}}$ then yields $A^{-1} \xrightarrow {\,p\,}G^{-1}$, $A^{-1}\tilde{\textbf{z}} \xrightarrow {\,p\,}G^{-1}\textbf{g}$, and $\sqrt{\tilde{\textbf{z}}^\top A^{-1}\tilde{\textbf{u}}} \xrightarrow {\,p\,}\sqrt{\textbf{g}^\top G^{-1}\boldsymbol{\mu }_z}$, respectively. Since the denominator converges to the strictly positive constant $\sqrt{\textbf{g}^\top G^{-1}\boldsymbol{\mu }_z}$, Slutsky’s theorem concludes the proof. □

### Theorem 5

($O_p(N^{-1/2})$ convergence rate) Under Assumptions 1–3, with *K* fixed, N>K, and $\textbf{g}^\top G^{-1}\boldsymbol{\mu }_z > 0$, there exists a constant M<∞, depending only on *C*, *K*, $\Vert G^{-1}\Vert _F$, and $\textbf{g}^\top G^{-1}\boldsymbol{\mu }_z$, such that

$$ \left| c_N-c^*\right| = O_p(N^{-1/2}) $$

### Proof

Write $\tilde{\textbf{z}} = \bar{\textbf{z}}/(N-1)$, $\tilde{\textbf{u}} = \textbf{z}/(N-1)$, and $f(A,\tilde{z},\tilde{u})$ for the $c_N$ map, so that $\textrm{OSMC}^* = f(G, \textbf{g}, \boldsymbol{\mu }_z)$ is the probability limit established in Theorem 4.

Since all summands are i.i.d. and bounded, Hoeffding’s inequality gives exponential tail bounds for each entry of *A*, $\tilde{\textbf{z}}$, and $\tilde{\textbf{u}}$. A union bound over all $K^2 + 2K$ entries gives

F11 $$\begin{aligned}  &   \Vert A - G\Vert _F \;=\; O_p(N^{-1/2}), \quad \Vert \tilde{\textbf{z}} - \textbf{g}\Vert \;=\; O_p(N^{-1/2}), \nonumber \\  &   \quad \Vert \tilde{\textbf{u}} - \boldsymbol{\mu }_z\Vert \;=\; O_p(N^{-1/2}), \end{aligned}$$

with constants depending on *C* and *K*.

Let $\lambda _{\min }(G) > 0$ denote the smallest eigenvalue of *G*, which is positive by Assumption 3. On the event $\mathcal {E}_N = \{\Vert A - G\Vert _F \le \lambda _{\min }(G)/2\}$, which has probability tending to one by (F11), *A* is itself invertible and the identity $A^{-1} - G^{-1} = A^{-1}(G - A)G^{-1}$ gives

$$ \Vert A^{-1} - G^{-1}\Vert _F \;\le \; \Vert A^{-1}\Vert _F\,\Vert G - A\Vert _F\,\Vert G^{-1}\Vert _F. $$

On $\mathcal {E}_N$, all eigenvalues of *A* are at least $\lambda _{\min }(G)/2 > 0$, so $\Vert A^{-1}\Vert _F \le 2/\lambda _{\min }(G)$, and similarly $\Vert G^{-1}\Vert _F \le \sqrt{K}/\lambda _{\min }(G)$. Substituting,

$$ \Vert A^{-1} - G^{-1}\Vert _F \;\le \; \frac{2}{\lambda _{\min }(G)^2}\,\Vert A - G\Vert _F \;=\; O_p(N^{-1/2}), $$

where the last equality uses (F11) and the constants are absorbed into *M*.

Since *f* is continuously differentiable in a neighborhood of $(G, \textbf{g}, \boldsymbol{\mu }_z)$, a first-order Taylor expansion gives

$$\begin{aligned}  &   f(A, \tilde{\textbf{z}}, \tilde{\textbf{u}}) - f(G, \textbf{g}, \boldsymbol{\mu }_z) \;=\; \nabla f\big |_{(G,\textbf{g},\boldsymbol{\mu }_z)} \cdot \begin{pmatrix} A - G \\ \tilde{\textbf{z}} - \textbf{g} \\ \tilde{\textbf{u}} - \boldsymbol{\mu }_z \end{pmatrix} \\  &   \;+\; o_p\!\left( \left\| \begin{pmatrix} A - G \\ \tilde{\textbf{z}} - \textbf{g} \\ \tilde{\textbf{u}} - \boldsymbol{\mu }_z \end{pmatrix}\right\| \right) . \end{aligned}$$

The gradient ∇f evaluated at the population point has finite norm, depending on $\lambda _{\min }(G)^{-1}$ (through $G^{-1}$) and $(\textbf{g}^\top G^{-1}\boldsymbol{\mu }_z)^{-1/2}$ (through the denominator of *f*), both of which are finite by Assumption 3 and the positivity condition. Combined with (F11), the linear term is $O_p(N^{-1/2})$ and the remainder is $o_p(N^{-1/2})$, giving $\left| c_N-c^*\right| = O_p(N^{-1/2})$. □

### Remark 6

(Bernstein polynomial features) A natural choice satisfying all three assumptions is the Bernstein basis on [0, 1], with *K* basis functions. Since each basis function maps [0, 1] into [0, 1], Assumption 2 holds with C=1. Assumption 3 holds whenever the distribution of *x* has support of positive length in [0, 1], since the Bernstein basis spans the same space as the polynomials of matching degree, and no non-zero polynomial can vanish almost everywhere under that condition. The results hold for any compact interval [*a*, *b*] by a rescaling of *x*, with *C* remaining bounded.

## Footnotes

1 For a common illustration of this problem, consider points that lie uniformly on a *d*-dimensional hypercube of side length (1+ϵ)r. In this setup, only a proportion of $(1+\epsilon )^{-d}$ data points lie in the central inner hypercube of side length *r*, with the rest of the points lying in the outer shell. This demonstrates that the distance between data points becomes unsuitable for a meaningful description of the structure of high-dimensional data sets.

2 Using a QR-decomposition algorithm, we can express X=QR, where *Q* is an orthogonal matrix and *R* is an upper-triangular matrix. Then, we remove any feature *i*, where $|R_{i,i}| < \epsilon$ for some small ϵ>0.

3 If $\mathcal {F}$ is chosen to be the class of all functions, then the solution to the above problem can easily be shown to have the trivial solution $\phi (V_1) = \frac{V_2-\mathbb {E} \left[ V_2 \right] }{\text {Var}(V_2)}$.

4 In its most usual form it says that for any real continuous function *f* on the interval $\left[ a,b \right]$ and $\epsilon > 0, \exists$ a polynomial function *g* such that $\forall x \in \left[ a,b \right]$, we have |f(x)-g(x)|<ϵ.

5 This is due to PCA consisting of a covariance estimation step with complexity $O(nd^2)$ and a spectral decomposition step of complexity $O(d^3)$.

6 Note that this is a pattern for this specific toy data set and not a general pattern.

7 Alternatively, we can take a weighted average of the forecasts of each model, but the performance was inferior.

8 We use a significance level of 0.001. We consider a specific lag effect significant if the confidence interval of either the ACF or the PACF is above or below zero. It is important to note that in some cases, the ACF and PACF values can fail to identify the need of an ARIMA model. However, including this criterion can make the algorithm significantly faster, when forecasting targets like price returns.

9 To avoid look-ahead bias, the steps at lines 1, 3, 4, 6, 7, 8, 11, and 14 of Algorithm 1 use only observations available up to the training cutoff $L_0$. During the online phase, predictions at time t (line 16) are generated using observations from t-L up to t. The OLS model is subsequently re-fitted using only observations from t-L up to t, while the steps at lines 17-19 perform one-step updates of the previous states. Hence, neither prediction nor model fitting uses observations from future time-steps.

10 The returns considered are adjusted for splits and dividends.

11 The industry portfolio returns and the daily market factors are taken directly from the Kenneth R. French library [49]. Based on a market portfolio, the three factors include small minus big (SMB) based on firm size, high minus low (HML) based on the book-to-market ratio, and the market excess return of the portfolio.

12 Using the Sharpe Ratio significance test of [48]. This is based on a generalization of the t-test, that accounts for the non-normality of returns, by taking into account the kurtosis and skewness of the P&L distribution.

13 Instead of $d_{t}^\text {min}$, one could employ the average distance $\bar{d}_t = \frac{\sum _{j=t-L}^{t-1} d_{j,t}}{t}$.
